# Compositional and structural analysis of selected chromosomal domains from *Saccharomyces cerevisiae*

**DOI:** 10.1093/nar/gkt891

**Published:** 2013-10-06

**Authors:** Stephan Hamperl, Christopher R. Brown, Ana Villar Garea, Jorge Perez-Fernandez, Astrid Bruckmann, Katharina Huber, Manuel Wittner, Virginia Babl, Ulrike Stoeckl, Rainer Deutzmann, Hinrich Boeger, Herbert Tschochner, Philipp Milkereit, Joachim Griesenbeck

**Affiliations:** ^1^Universität Regensburg, Biochemie-Zentrum Regensburg (BZR), Lehrstuhl für Biochemie III, 93053 Regensburg, Germany and ^2^Department of Molecular, Cell, and Developmental Biology, University of California Santa Cruz, Santa Cruz, CA 95064, USA

## Abstract

Chromatin is the template for replication and transcription in the eukaryotic nucleus, which needs to be defined in composition and structure before these processes can be fully understood. We report an isolation protocol for the targeted purification of specific genomic regions in their native chromatin context from *Saccharomyces cerevisiae*. Subdomains of the multicopy ribosomal DNA locus containing transcription units of RNA polymerases I, II or III or an autonomous replication sequence were independently purified in sufficient amounts and purity to analyze protein composition and histone modifications by mass spectrometry. We present and discuss the proteomic data sets obtained for chromatin in different functional states. The native chromatin was further amenable to electron microscopy analysis yielding information about nucleosome occupancy and positioning at the single-molecule level. We also provide evidence that chromatin from virtually every single copy genomic locus of interest can be purified and analyzed by this technique.

## INTRODUCTION

Chromatin is dynamic and changes in its composition and structure depending on the functional state of the respective genomic region. The basic repeating unit of chromatin, the nucleosome, is composed of 147 basepairs (bp) of DNA wrapped around a core of eight histone proteins [reviewed in (1)]. Nucleosomes are not uniform because histone variants and different posttranslational covalent modifications establish distinct signatures in the chromatin landscape [reviewed in (2)], which is further characterized by the specific association of other protein factors with genomic regions. Most of our current knowledge regarding chromatin composition at defined genomic loci is based on chromatin immunoprecipitation (ChIP) [reviewed in (3)]. ChIP is helpful to map DNA interactions of known chromatin components but requires *a priori* knowledge for the selection of candidate proteins.

Various strategies have been used to isolate and analyze the composition of defined chromosomal domains to derive a description of chromatin at selected genomic loci (4–11). Most of these attempts suffered from low recovery or insufficient purity of the isolated material, making downstream analyses difficult. Recently, detailed information about protein composition at telomeres in human cell lines and in *Drosophila* was obtained by a method called PICh for Proteomics of Isolated Chromatin segments (12,13). Another report introduced chromatin affinity purification to investigate the proteome and posttranslational histone modifications at a single-copy locus in *S. cerevisiae* (hereafter called yeast) (14). Like PICh, chromatin affinity purification is carried out with formaldehyde crosslinked material under denaturing conditions and does not provide a source to native chromatin for functional and structural analysis. Two other studies in yeast analyzed the protein composition of centromere bound kinetochores or replication-dependent changes in post-translational histone modifications on multicopy plasmids (15,16). It remains unclear, however, whether chromatin on multi-copy plasmids fully reflects chromatin at chromosomal loci.

**Figure 1.**
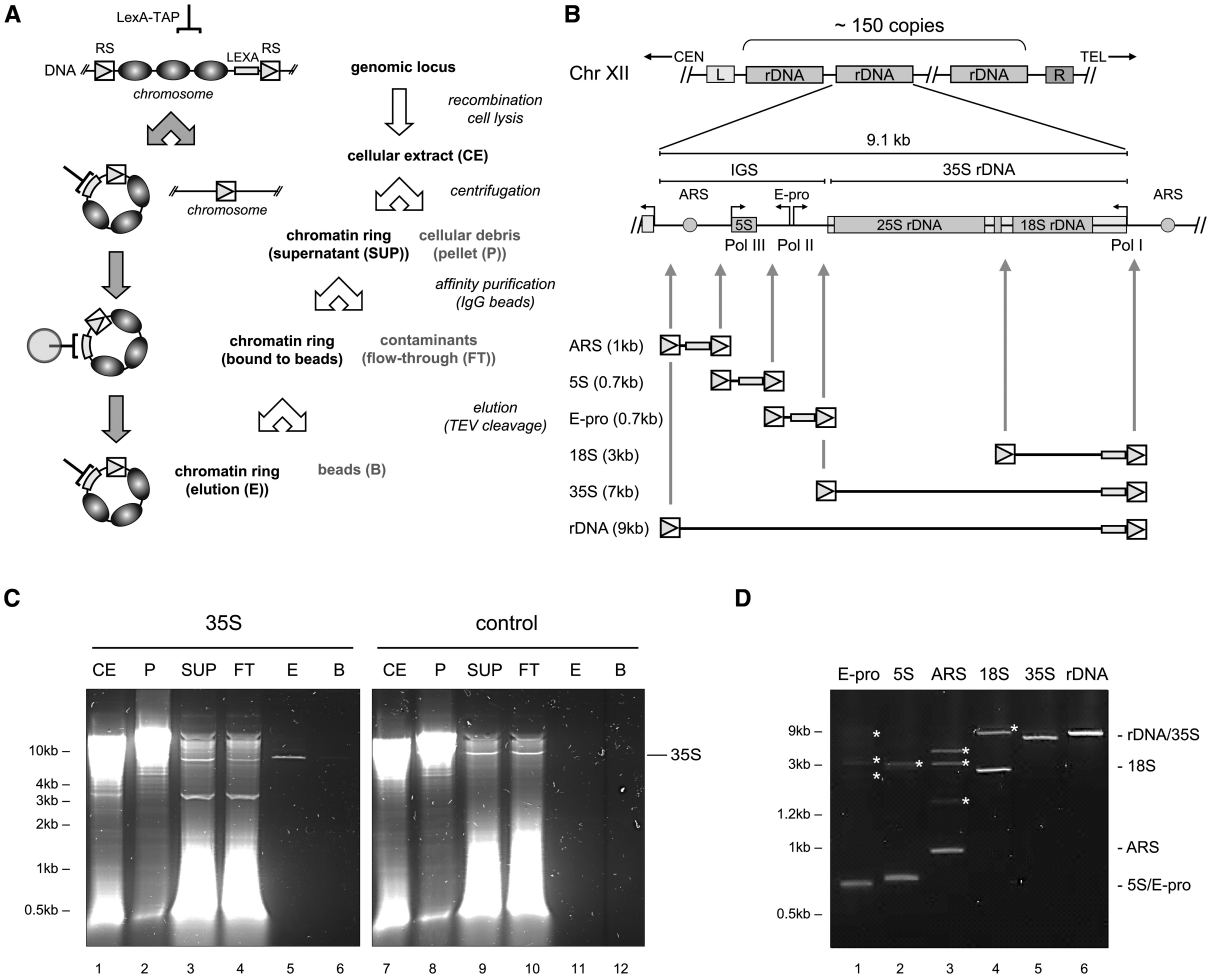
Distinct domains of the rDNA locus can be purified from yeast. (**A**) Purification of chromosomal domains. LEXA, cluster of LexA DNA binding sites; RS, sequences for site specific recombination; LexA-TAP, recombinant LexA fusion protein; filled ovals, chromatin components; filled circle, IgG coated magnetic beads. (**B**) Genetic manipulation of the rDNA locus. The rDNA locus on chromosome XII is depicted on the top. CEN, centromere; TEL, telomere; 35S, 25S, 18S, 5S rRNA coding regions; IGS, intergenic spacer region; ARS, autonomous replication sequence (gray circles); E-pro, expansion promoter; black arrows, transcription start sites used by Pol I, II, III; gray arrows; insertion sites of RS elements and LexA DNA binding sites; numbers in parentheses represent sizes of the different domains. (**C**) DNA analysis of samples of a chromatin domain purification and of a control purification. Yeast strains y2381 (35S) and y2378 (control), carrying the 35S rRNA gene domain flanked by RS sites, or lacking recombination sites, respectively, were subjected to the purification procedure. DNA was isolated from samples CE, SUP, P, FT, E and B (Figure 1A), SacII digested and analyzed in a 1% agarose gel stained with SYBR® Safe (Life technologies). The LexA bait protein was purified from 10^11^ cells and 0.7% (CE, P), 2.5% (SUP, FT) and 8.3% (E, B) from the respective samples were analyzed. Positions of DNA size markers, and of the linearized 35S rRNA gene ring DNA (35S) are indicated. (**D**) DNA analysis of purified rDNA chromatin domains. Chromatin domains were purified from yeast strains y2384 (E-Pro), y2379 (5S), y2383 (ARS), y2380 (18S), y2381 (35S) and y2382 (rDNA). DNA was isolated from TEV eluates, digested with NcoI (E-pro, 5S, ARS) or SacII (18S, 35S, rDNA) and analyzed as described in the legend to Figure 1A. The LexA bait protein was purified from 10^11^ cells, and 5% of the respective samples were analyzed. Positions of DNA size markers and of restriction fragments of the individual rDNA segments are indicated. Asterisks mark restriction fragments from higher-order recombination products of the respective domain.

Here, we describe a protocol for the isolation of native chromosomal domains from yeast. We built on a previously established technique based on site specific recombination at chromosomal loci, which had been tagged with a cluster of LexA DNA binding sites (Figure 1A) (7). After recombination, chromosomal domains were released in the form of a chromatin ring and could be isolated via a co-expressed recombinant LexA protein fused to a tandem affinity purification (TAP) tag (17). While this previously established technique allowed isolation of chromosomal domains (7), the chromatin preparations were of insufficient purity to perform some of the intended analyses. We therefore developed a new purification strategy now enabling compositional and structural analyses. First, the approach was applied to the multicopy ribosomal DNA (rDNA) gene cluster, which consists of 150–200 tandem repeats on chromosome XII (Figure 1B) [reviewed in (18)]. The rDNA locus contains the 35S ribosomal RNA (rRNA) gene, a bi-directional promoter (E-pro) and the 5S rRNA gene transcribed by RNA polymerases (Pols) I, II and III, respectively, as well as an autonomous replication sequence (ARS). These functional genomic elements could be individually purified and analyzed. Finally, isolation of a chromatin domain containing the single copy *PHO5* gene and identification of co-purifying proteins by mass spectrometry (MS) suggested that this purification strategy will be applicable to any genomic locus of interest.

## MATERIALS AND METHODS

### Plasmids and yeast strains

Unless noted otherwise, standard techniques were used for cloning of plasmids and transformation of yeast cells (19,20). Complete lists of oligonucleotides, plasmids and yeast strains can be found in Supplementary Tables S1–S3.

#### Generation of yeast strains containing a modified endogenous rDNA locus

To generate pT30, oligonucleotides 1045 and 1046 were annealed and cloned into AflII digested plasmid K773 (pT28) (21). Plasmid K366 (pT2) (21) was digested SacII and DraIII, and the resulting 8.812 bp fragment was ligated with the 1.507 bp SacII and Dra III fragment of pT30, yielding pT32. Plasmid K450 (pKM4) (21) was digested with BamHI and SbfI and cloned into BamHI and SbfI digested YEplac195 (22) yielding plasmid K451 (pKM5). A 4.439 bp MluI-SbfI fragment of pT32 was inserted into the 10.203 bp MluI-SbfI backbone of K451 (pKM5), yielding K673 (pT34). Plasmid K375 (pT11) was digested with PstI and BamHI, and the resulting 4.526 bp fragment was cloned into the BamHI and PstI digested backbone of K673 (pT34) resulting in plasmid K674 (pT36).

For construction of plasmids containing a modified intergenic spacer (IGS) region of the rDNA locus, a BamHI/NotI fragment of pNOY373 (23) was cloned into BamHI and NotI digested pBluescript KS (Stratagene) resulting in plasmid K1560 (pBluescript BamHI NotI 5S). A NotI/BsrGI fragment containing the E-pro region, a BseRI/SphI fragment containing the 5S rDNA gene and a SphI/BamHI fragment containing the ARS element from K1560 were blunted and cloned into HpaI and XhoI digested, blunted plasmid pM49.2 (24) yielding K1578 (pUS6), pMW5a and pUS1, respectively. The resulting plasmids were digested with BamHI and PstI for pUS6 and pMW5a or with BamHI and SbfI for pUS1, blunted and reinserted into NotI and BsrGI digested, blunted, BsrGI and SphI digested, blunted or SphI and BamHI digested, blunted K1560 yielding pUS9b, pBluescript_5S-RS and K1577 (pUS3), respectively. The self-complementary oligonucleotide 2629 was annealed and ligated with SacII restricted pUS9b to introduce a NotI restriction site yielding K2024 (pUS9b_NotI). pBluescript_5S-RS was digested with NotI and SmaI, and the resulting 2.158 bp fragment was cloned into NotI/SmaI digested pNOY373 yielding pNOY373_5S-RS. pUS9b_NotI was digested with NotI and BamHI and cloned into NotI and BamHI digested pNOY373 yielding pUS11. pUS3 was digested with NotI and EcoRI, and the blunted 2.197 bp fragment was ligated with NotI and BamHI digested blunted pNOY373 yielding pUS5. pNOY373_5S-RS was digested with SmaI and PstI, and the 9.457 bp fragment was cloned into PstI and BamHI-digested pT4 (21) yielding K1185 (pAG43). pUS5 was linearized with PstI and ligated with PstI linearized pT4 resulting in K2026 (pUS7). pUS11 was digested with BamHI and PstI and cloned into BamHI and PstI digested pT4 resulting in K2027 (pUS12).

Generation of plasmids K375, K389, K1185 and K1785 has been described in (21).

A previously described method was used to genetically modify the endogenous rDNA locus (23) such that each rDNA repeat contained the specific insertion of RS elements and LexA-binding sites. To this end, the plasmids K375 (pT11), K389 (pT25), K674 (pT36), K1185 (pAG43), K1785 (pT11-WT), K2026 (pUS7), K2027 (pUS12) were digested with SpeI. The resulting DNA fragments contained a wild-type rDNA repeat (K1785) or a modified rDNA repeat, in which either the ARS (K2026), the 5S rRNA gene (K1185), the E-pro region (K2027), the 18S rRNA coding sequence (K674), the 35S rRNA gene (K389) or a complete rDNA repeat (K375) are flanked by RS elements and include LexA-binding sites. These fragments also contained sequences for integration and expansion of the modified rDNA repeats as described earlier (23). After transformation into yeast strain NOY989, mutant clones containing a re-expanded modified rDNA locus were selected as described (23).

None of these yeast strains had detectable growth defects suggesting that the RS elements and LexA-binding sites integrated in the rDNA locus did not significantly affect rDNA transcription and ribosome production in the cell. We note that some of the strains containing insertions at or in the proximity of the NotI restriction site within the region 3′ of the 35S rRNA gene [present in pNOY373 (23)] had a lower rDNA copy number. A reduced copy number had also been reported in a strain where the NotI restriction site had been used to delete part of the rDNA enhancer region, before re-integration and re-expansion of the modified rDNA repeat (25).

#### Generation of plasmids for expression of R recombinase and LexA-TAP

A set of plasmids for inducible expression of R recombinase and constitutive expression of LexA-TAP was constructed. The *CYC1* or *TEF2* promoter regions, supporting either minimal or robust expression of the fusion protein, were amplified with primer pairs 2507, 2511 or 2508, 2512 from yeast genomic DNA. The PCR products were digested with XbaI and XhoI and cloned into XbaI and XhoI digested pBluescript KS yielding plasmids pBluescript_CYC1 and pBluescript_TEF2. The KpnI/XbaI fragment of plasmid pJSS3 (24) containing the coding sequence of LexA-TAP was cloned into KpnI and XbaI digested pBluescript_CYC1 and pBluescript_TEF2 resulting in pBluescript_CYC1 LexA-TAP and pBluescript_TEF2 LexA-TAP. The plasmids were digested with BamHI and KpnI and the resulting 2.734 or 2.848 bp fragments were blunted and cloned into SmaI linearized pB3 (24) yielding K2048 (pSH15) and K2049 (pSH17).

Another plasmid K929 (pKG7) allows for inducible expression of R recombinase and LexA-TAP both under control of the bidirectional *GAL1-10* promoter. To this end, plasmid K356 (pJSS3) (24) was linearized with BssHII, and the resulting 7.891 bp fragment was blunted and ligated with SmaI linearized plasmid K355 (pB3) yielding K363 (pR2). K363 was digested with SpeI, and the resulting 11.239 bp fragment was ligated yielding K929 (pKG7). The plasmids K2048 (pSH15), K2049 (pSH17) and K929 (pKG7) allow ectopic expression of R recombinase under control of the inducible *GAL1-10* promoter and expression of the LexA-TAP protein under control of the constitutive *CYC1*, *TEF2* or inducible *GAL1-10* promoter, respectively. The transformed plasmids have to be maintained in the yeast cell, which is accomplished by a selectable *LEU2* marker gene present on all the expression plasmids. However, cells must be grown in minimal medium lacking leucine to obtain uniform cell populations including the plasmid.

To conduct the chromatin preparations from yeast cells grown in full medium, the expression cassettes for R recombinase and LexA-TAP were flanked with homologous sequences of the yeast *URA3* gene. To this end, the *URA3*-coding sequence was amplified with primer pair 2686, 2687 from yeast genomic DNA. The PCR product was digested with KpnI and SacII and cloned into KpnI and SacII digested pBluescript KS yielding K2051 (pBluescript_URA3). Plasmids pSH15 and pSH17 were digested with BssSI, and the resulting 9.314 or 9428 bp fragments were blunted and cloned into StuI linearized plasmid K2051 yielding K2054 (pSH23) and K2052 (pSH21). K2052 (pSH21) was digested with HindIII and PflFI, blunted and religated yielding K2053 (pSH22). The plasmids K2052 (pSH21) and K2054 (pSH23) allow genomic integration of the expression cassettes for R recombinase under control of the inducible *GAL1-10* promoter and the LexA-TAP protein under control of the constitutive *TEF2* or *CYC1* promoters, respectively. Plasmid K2053 (pSH22) allows genomic integration of the expression cassette for R recombinase under control of the *GAL1-10* promoter without LexA-TAP expression. The plasmids were digested with SbfI, and the resulting DNA fragments were transformed in the yeast cells allowing the stable chromosomal integration of the expression cassette at the endogenous *URA3* locus by homologous recombination. Mutant clones for positive integration were selected on SCD-LEU plates for the *LEU2* marker present on the expression cassettes.

#### Generation of yeast strains expressing MNase fusion proteins

Strains expressing yeast proteins with a C-terminal MNase carrying a triple hemagglutinin (3 × HA) epitope from their chromosomal location were generated as described previously (26). Expression of the MNase fusion protein was verified by western blot analysis with antibody 3F10 (Roche) recognizing the C-terminal 3 × HA-tag of the fusion proteins [data not shown, see also (26)]. None of the strains expressing MNase fusion proteins showed an obvious growth phenotype (data not shown).

### Affinity purification of chromatin domains

Yeast cells competent for excision of chromatin domains by R recombinase were cultivated overnight at 30°C in Yeast extract-peptone-raffinose or selective complete raffinose medium lacking leucine (SCR-LEU). For rDNA ring purifications, we used yeast strains in which the LexA-TAP protein was constitutively expressed at moderate levels controlled by the *TEF2* promoter as opposed to the strong *GPD* promoter used in earlier studies (7,24,27). For purification of *PHO5* gene rings LexA-TAP levels were further reduced by using the weak *CYC1* promoter for expression of the protein (27). Reducing the cellular levels of the fusion protein led to a lower background with chromatin from sheared bulk genomic DNA in the preparations. Recombination was induced at a cell density of 5–7 × 10^7^ cells/ml (OD_600_ of 0.8–1.0) by adding galactose to a final concentration of 2% (w/v). Cells were grown for an additional 1.5 h at 30°C before harvesting to allow proper expression of R recombinase and formation of chromatin rings. After induction, cells were harvested by centrifugation (10 min, 9000 × *g* at 4°C), yielding a wet weight between 3 and 4 g per liter of cell culture. After washing twice with water, cells were pelleted in sealed 20 ml syringes by centrifugation (5 min, 3000 × *g* at 4°C). Supernatants were decanted, syringes were unsealed and cells were extruded into liquid nitrogen. The resulting ‘cell spaghetti’ were stored at −80°C until use. A commercial coffee grinder (TEFAL, Prep`line) was pre-cooled by grinding 30–50 g of dry ice twice. The resulting powder of dry ice was discarded. Appropriate amount of frozen cells (3–4 g for rDNA ring purification or 18–40 g for *PHO5* gene ring purification) were mixed with ∼60 g of dry ice in the coffee mill. Grinding was repeated three times for 1 min with short intervals in between grinding to prevent heating of the coffee mill motor. Shaking of the coffee mill while grinding prevented the dry ice–cell powder from sticking to the inside wall of the grinding chamber. The fine powder of ground yeast can be stored at −80°C.

Protein A-immunoglobulin G (IgG) affinity purification was performed as described previously (28,29) with minor modifications. After evaporation of dry ice, the powder was dissolved in 0.75 ml of cold buffer MB [20 mM Tris–HCl (pH 8), 200 mM KCl, 5 mM MgAc, 0.5% Triton X-100, 0.1% Tween-20, 1 mM DTT] with 1× Protease inhibitors (0.17 mg/ml PMSF and 0.33 mg/ml benzamidine) per 1 g of ground yeast cells. The cell lysate was cleared from cell debris by centrifugation for 30 min at 16.000 *g* and at 4°C. From 1 g of yeast cells, ∼1 ml of cell lysate with a protein concentration of 15–20 mg/ml was obtained. To generate the affinity resin, rabbit IgGs (Sigma) were added to epoxy-activated magnetic beads (BcMag™, Bioclone Inc.) in a ratio of 0.17 mg IgGs/mg of beads according to the manufacturers instruction. The IgGs coupled to magnetic beads were equilibrated with buffer MB before use. For the purification of rDNA rings, 7.5 mg of magnetic beads with coupled IgGs were added to a total amount of 3 ml of cell lysate. For the purification of *PHO5* gene rings 3.75 mg of magnetic beads with coupled IgGs were added to a total amount of 15 ml of cell lysate. The cell lysate-bead suspension was incubated on a rotating wheel for 1 h at 4°C. Beads were washed five times with 750 μl of cold buffer MB with 1× Protease inhibitors per 3.75 mg of magnetic beads with coupled IgGs. Between each washing step, the beads were gently rotated for 10 min. Finally, the beads were washed with 750 μl of cold buffer MB without Protease inhibitors per 3.75 mg of magnetic beads with coupled IgGs. Chromatin rings were eluted by proteolytic cleavage for 2 h (or overnight) at 4°C with 10 μg 6×His-tagged recombinant Tobacco Etch Virus (TEV) protease in a total volume of 100 μl per 3.75 mg of magnetic beads with coupled IgGs. The supernatant was transferred to a new microtube, and residual chromatin circles were washed from the beads with another 100 μl of buffer MB and combined with the eluate.

To deplete the 6xHis tagged TEV protease from the purified rDNA chromatin rings, 20 μl of Ni-NTA beads (Qiagen) equilibrated with buffer MB were incubated with the eluate in a sealed 1.5 ml column (Bio-Spin, BioRad) for 30 min at 4°C on a rotating wheel. The column was unsealed, and the sample was transferred to a new microtube. This step removed virtually all of the recombinant TEV protease, which was important for subsequent MS analysis.

For *PHO5* chromatin rings eluting from the IgG beads after TEV protease cleavage, the volume was adjusted to a total volume of 400 μl with buffer MB and supplemented to a final concentration of 2 mM CaCl_2_. The elution sample was applied to 100 μl of calmodulin sepharose beads (Stratagene) equilibrated with buffer MB containing 2 mM CaCl_2_ for at least 2 h. Beads were incubated for 1 h at 4°C and washed 4× with 1 ml of buffer CWB [20 mM Tris–HCl (pH 8), 300 mM KCl, 5 mM MgAc, 0.5% Triton X-100, 0.1% Tween-20, 2 mM CaCl2, 1 mM DTT]. Chromatin rings were eluted from calmodulin beads by gravity flow with 100 μl of buffer CEB [20 mM Tris–HCl (pH 8), 200 mM KCl, 5 mM MgAc, 1 mM EDTA, 10 mM EGTA, 0.5% Triton X-100, 0.1% Tween-20, 1 mM DTT]. After sealing the column, 200 μl of buffer CEB were added to the beads, and the slurry was incubated for 20 min at 4°C under shaking at 1000 rpm in a IKA-Vibrax shaker. Buffer CEB was collected by gravity flow before another 100 μl of buffer CEB were applied to wash residual chromatin rings from the beads. All elution steps were combined yielding a total volume of 400 μl.

### Blot analysis

Western blot analysis was performed according to standard procedures (30). Supplementary Table S4 contains a complete list of antibodies used for detection.

Southern blot analysis was performed as described (26). Supplementary Table S5 contains a complete list of hybridization probes.

### Analysis of histone modifications by MALDI TOF/TOF MS

Histones H3 and H4 were extracted from an SDS–polyacrylamide gel and analyzed as previously described (31) with minor modifications. After destaining the gel pieces with 0.1 M ammonium bicarbonate in 30% high performance liquid chromatography-grade acetonitrile, samples were washed twice with 70% high performance liquid chromatography-grade methanol and acylated with 0.71 M propanoic anhydride in 70% methanol. Proteins were then digested with trypsin (Roche Applied Science) according to manufacturer’s instructions, and 0.5–1 µl of aliquots were spotted in triplicate onto a stainless steel MALDI target plate, allowed to dry, overlaid with 0.5 µl of freshly prepared, ice-cold 5 mg/ml alpha-cyano-4-hydroxy cinnamic acid (for mass spectrometry, Fluka) in 50% acetonitrile/0.3% TFA, dried again and analyzed by MALDI-TOF/TOF in a 4800 instrument from Applied Biosystems, operated according to manufacturer’s instructions. Full MS spectra were acquired in the reflector modus, with m/z from 700 to 2000 (H4) or 700 to 2500 (H3); focus on 1500 (H4) or 1600 (H3), and 50 × 50 spectra were acquired per spot. For the tandem MS/MS spectra, the peptide 4–17 of H4 was fragmented in PSD modus, whereas the peptides of H3 were fragmented with collision-induced dissociation; in all the cases, the isolation window width was 1.0 Da; an inclusion list was used for parent ion selection (tolerance 0.3 Da), and 85 × 45 shots per spectra were acquired. For all MS and MS/MS spectra, the laser intensity was manually adjusted for optimized S/N.

Spectra were processed with mMass 5.2.0 (32,33). After recalibration by using signals of the histones (peptides 46–55 and 79–92 for H4 and peptides 64–69 and 27–40 unmodified for H3), the relative proportion of a given modification for the peptides of interest was calculated by dividing the absolute intensity of the signal corresponding to that modification through the sum of the intensities for all the signals corresponding to any modified species from the same peptide. The results for all the MALDI replicas of each measurement were averaged.

### Comparative iTRAQ MALDI TOF/TOF MS analysis

Proteins present in the elution samples of chromatin ring preparations (see earlier in the text) were precipitated by methanol/chloroform (34). Digestion of the proteins with trypsin, iTRAQ labeling and quantitative MALDI MS analysis were essentially performed as described elsewhere (29). The processed data set is presented in Supplementary Data Sets 1 and 2.

### Chromatin endogenous cleavage and ChIP

For chromatin endogenous cleavage (ChEC) experiments, yeast strains expressing the MNase fusion proteins from their endogenous genomic location were grown in yeast peptone dextrose at 30°C to a final OD_600_ of 0.5. ChEC analyses were performed as previously described (26).

ChIP was performed as described elsewhere (35). Primer pairs used for amplification are listed in Supplementary Table S1. Data were collected with a Rotor-Gene 3000 system (Corbett Research) and analyzed using the comparative quantitation module of the system software. Retention of specific DNA fragments was calculated as a percentage of total input DNA. The mean values and error bars are derived from at least three independent ChIP experiments analyzed in triplicate quantitative PCR reactions.

### Restriction endonuclease digestion analysis of chromosomal and purified chromatin domains

For restriction endonuclease digestion of chromosomal and affinity purified chromatin domains, crude nuclei were prepared as described in (21). The 5S rRNA gene chromatin rings were purified as detailed earlier in the text. An aliquot of the crude nuclei corresponding to 2 × 10^8^ cells or 1.25% of the TEV elution were adjusted to a final volume of 100 μl with the buffer recommended for the respective restriction enzyme (NEB). Digestion was performed for 60 min at the optimal reaction temperature using different amounts of the respective restriction endonuclease (10 or 50 U for nuclei and 2 or 20 U for the purified chromatin ring). The reaction was terminated by adding one volume of IRN buffer [50 mM Tris–HCl (pH 8), 20 mM EDTA, 500 mM NaCl]. Samples were treated with RNase A at a final concentration of 0.33 mg/ml for 1 h at 37°C. Proteinase K and SDS were added to a final concentration of 0.33 mg/ml and 0.5%, and incubation was continued for 1 h at 56°C. After phenol/chloroform extraction, DNA was precipitated with ethanol in the presence of 40 μg of glycogen as a carrier. The DNA was suspended in 20 μl of H_2_O, and DNA molecules were linearized by restriction enzyme digestion overnight at 37°C in a final volume of 30 μl with 20 U of PvuI and SphI (nuclei) or 20 U NcoI (chromatin rings), respectively. The DNA samples were subjected to indirect endlabeling Southern blot analysis.

As a control, each restriction enzymatic digestion was also performed with samples deproteinized by phenol/chloroform extraction. The DNA was almost completely digested in all cases (not shown).

### Trimethylpsoralen crosslinking of rDNA chromatin rings and preparation of DNA for electron microscopy analysis

Crosslinking was performed as described (36) with some modifications. Purified chromatin rings in 5 ml of buffer CEB were placed onto a 10-cm petri dish floating on an ice water slurry; after addition of 0.05 volumes of 400 µg/ml trimethylpsoralen in ethanol the sample was incubated in the dark on ice for 5 min. Samples were positioned 5-cm away from five 366-nm ultraviolet bulbs in a Stratalinker 2400 (Stratagene) and then irradiated for 5 min. Addition of psoralen, incubation in the dark, and irradiation were performed a total of seven times for each sample. After treatment with RNase A (at a final concentration of 0.33 mg/ml for 2 h at 37°C), Proteinase K and SDS were added to a final concentration of 0.33 mg/ml and 0.5% and incubation was continued for 4 h at 55°C. DNA was extracted with phenol/chloroform and precipitated. DNA was suspended, digested with NcoI, purified using a DNA Clean and Concentrator Kit (ZymoResearch) and eluted from the column with 8 µl of TEN (30 mM Tetraethylammoniumchloride, 20 mM EDTA, 10 mM NaCl). Denaturing, spreading, staining with uranyl acetate and rotary metal shadowing were performed as previously described (36).

### Electron microscopy

Images were taken on a JEOL 1230 electron microscope at 120 keV at 20 000× magnification. Images were processed and analyzed in ImageJ (37).

## RESULTS

### Distinct chromatin domains of the rDNA can be purified from yeast

Yeast strains were constructed in which either the ARS, the 5S rRNA gene, the E-pro region, the 35S rRNA gene, the 18S rRNA coding sequence or a complete rDNA repeat are flanked by RS elements and include LexA-binding sites (Figure 1B). The cells also contained a chromosomally integrated cassette for constitutive expression of a LexA-TAP fusion protein. Chromosomal integration of the expression cassette as well as reducing the LexA-TAP expression levels by the choice of an appropriate yeast promoter were major differences to the previous purification protocol (7) (see ‘Materials and Methods’ section for details). After recombination, cells were lysed, and the whole-cell extract was subjected to a single-step affinity purification protocol using IgG coupled to magnetic beads (28). This change in the purification strategy proved to be important to obtain chromatin preparations, that are suitable for MS and electron microscopy (EM) analyses. Figure 1C shows a representative nucleic acid analysis of different samples taken during the purification of a chromosomal 35S rDNA domain (Figure 1C, lanes 1–6). The 35S rDNA domain was efficiently eluted under native conditions following TEV protease cleavage between the C-terminal protein A moiety and the calmodulin binding peptide (CBP) of LexA-TAP (Figure 1C, lane 5). Similarly, the E-pro-, 5S-, ARS-, 18S-regions as well as the entire rDNA repeat could be purified (Figure 1D; Supplementary Figure S1). No nucleic acids were detected in the control purification from an isogenic yeast strain lacking sites for recombination and LexA binding (Figure 1C, lane 11). In some preparations, larger DNA fragments were detected in addition to the fragment expected for the specific domain (Figure 1D, labeled by asterisks). These fragments originated from higher-order recombination products as confirmed in Southern blot analysis (Supplementary Figure S1). Around 50–500 fmoles of purified domains were obtained from one liter of exponentially growing yeast culture (∼10^11^ cells) corresponding to 2–13% of total cellular rDNA domains (Table 1). In the final elution fraction the specific chromosomal domains were present in a 4000-fold to 30 000-fold excess over another unrelated single copy gene locus as determined by quantitative PCR (Table 1).

**Table 1.** Recovery of chromosomal domains on extraction and affinity purification

Locus	rDNA				PHO5
Domain	E-pro	5S	ARS	35S	Gene
Recombination efficiency	55%	61%	66%	n.d.	74%
Cell number	10^11^	10^11^	10^11^	10^11^	1.5x10^12^
Amount of domain [fmoles]					
Extraction					
Cellular extract (CE)	1234	3521	4235	2988	1678
Pellet (P)	642	2320	1716	2102	439
Supernatant (SUP)	611	1398	2612	446	632
IgG magnetic beads					
Flow through (FT)	134	717	757	131	224
**Elution (E)**	**161**	**248**	**534**	**53**	**191**
Beads (B)	37	26	97	4	16
Calmodulin sepharose					
Flow through (FT)					119
**Elution (E)**					**50**
Beads (B)					6
Recovery					
Fraction of CE in IgG magnetic beads elution	**13%**	**7%**	**13%**	**2%**	**11%**
Fraction of CE in Calmodulin sepharose elution					**3%**
Excess over PDC1 locus					
In the elution from IgG magnetic beads	8300	32 460	18 896	4259	16 845
In the elution from Calmodulin sepharose					146 702

The absolute molar amounts of the DNA of the respective chromosomal domain and the single copy were determined by quantitation of the Southern blots shown in Supplementary Figure S1, and quantitative PCR. The recovery of the individual chromosomal domain in the elution fraction of the affinity purification is the quotient of the amount of recombined locus in the elution divided by the amount of recombined locus in the cellular extract. The excess of the purified domain over the *PDC1* locus is the quotient of the amount of the respective domain divided by the amount of *PDC1* locus in the indicated fractions as determined by quantitative PCR. The efficiency of recombination is the quotient of the amount of recombined locus divided by the amount of non-recombined locus determined by quantitation of the Southern blots shown in Supplementary Figure S1. Bold values indicate absolute amounts and percentage of chromatin domains recovered in the elution fractions.

### rDNA subdomains exhibit distinct histone modification patterns

**Figure 2.**
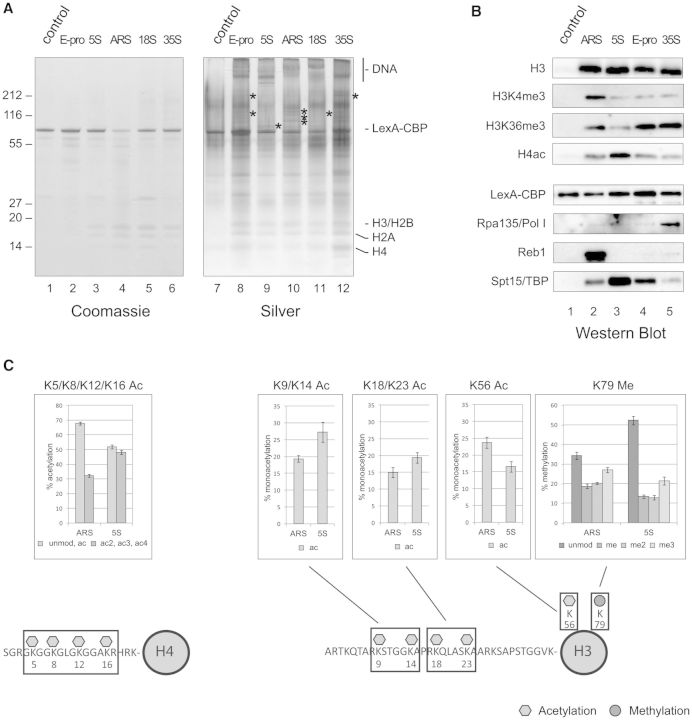
rDNA subdomains carry distinct histone modification patterns. (**A**) Analysis of proteins co-purifying with rDNA chromatin domains. Proteins co-purifying with LexA-TAP from yeast strains y2378 (control), y2384 (E-Pro), y2379 (5S), y2383 (ARS), y2380 (18S) and y2381 (35S) were separated in an SDS–PAGE gradient gel (4–12%) stained with colloidal Coomassie blue (lanes 1–6), or with silver (lanes 7–12). The LexA bait protein was purified from 10^11^ cells and 50% of the respective samples were analyzed. Positions of protein size markers, and of the LexA fusion protein (LexA-CBP), the core histones (H3/H2B, H2A, H4), as well as the ring DNA (visualized by silver stain) are indicated. Asterisks mark specific protein bands detected in purifications of the different rDNA domains. (**B**) Western blot analysis detecting proteins co-purifying with rDNA chromatin domains. Proteins co-purifying with LexA-TAP from yeast strains y2378 (control), y2383 (ARS), y2379 (5S), y2384 (E-Pro) and y2381 (35S) were separated in an 18% SDS–PAGE and subjected to western blot analysis with antibodies raised against covalent histone modifications and the indicated proteins. The LexA bait protein was purified from 10^11^ cells and 5% of the respective samples were analyzed. (**C**) Mass spectrometric analysis of histone modifications in ARS and 5S rRNA gene chromatin. Chromatin domains were purified from 2 × 10^11^ cells of yeast strains y1997[K2049] (5S) and y2267[K2049] (ARS), and half of the respective preparation was separated in an SDS–PAGE gradient gel (4–12%). Coomassie-stained histone bands were excised, and trypsin-digested peptides were subjected to MALDI TOF/TOF analysis. Spectra were quantified, and the results for a respective peptide are depicted in bar diagrams, as a percentage of the total amount of this peptide in the analysis. Average values and standard deviation are from two biological replicates. Cartoons below the graph show the relevant amino acid sequence of H4 and H3 with the lysine residues carrying the respective modification.

Proteins co-purifying with individual domains or in the control purification were analyzed by SDS–PAGE and Coomassie staining. The LexA fusion protein (LexA-CBP) was similarly enriched in all preparations (Figure 2A, lanes 1–6). Small proteins specifically co-purifying with the rDNA domains were identified as the canonical histones after excision from the gel and MS analysis (Figure 2A, lanes 2–5, Figure 2C). The post-translational modification state of histones was investigated by western blot analyses (Figure 2B). Similar amounts of unmodified histone H3 were detected in the chromatin preparations, but not in the control purification. H3 lysine 4 tri-methylation (H3K4me3) was enriched in the ARS domain purification, corresponding well with ChIP data in a previous report (38). Histone H3 lysine 36 tri-methylation (H3K36me3) was instead preferentially found in preparations of the E-pro and 35S rDNA domains. The level of acetylated lysine residues 5, 8, 12 and 16 within histone H4 (H4ac) was increased in purified 5S rDNA domains again in good correlation with ChIP experiments (39).

Post-translational covalent modifications of gel purified histone proteins in preparations of ARS and 5S rDNA subdomains were additionally assessed by MS confirming the increased acetylation within the histone H4 N-terminal residues 4–17 in the preparations containing purified 5S rDNA subdomain (Figure 2C). Monoacetylated H3 peptides including K9/K14 and K18/K23 were slightly enriched in the 5S purification compared with the ARS sample (K9/K14 Ac, K18/K23 Ac) correlating with previous ChIP results (39). H3K56 acetylation, playing a role in the regulation of replication-coupled nucleosome assembly (40,41), was enriched within the ARS preparation (Figure 2C, K56 Ac). We also quantified the relative amounts of unmodified, mono-, di- and tri-methylated peptides including K79 of histone H3 (Figure 2C, K79 Me). The percentage of unmodified K79 was significantly enriched in the 5S sample compared with the ARS chromatin purification. Histone H3 molecules associated with the ARS and 5S rRNA gene rings appeared to be hypomethylated at K79 when compared with the 18S rRNA gene domain or a preparation of bulk yeast chromatin (data not shown). This observation is also supported by previous ChIP analyses (42). We conclude that distinct histone modification patterns of purified rDNA domains are preserved after native purification and can be determined by MS.

### Specific chromatin components are enriched in purifications of rDNA subdomains

The silver stained gel in Figure 2A revealed specific protein bands present in individual chromatin preparations (Figure 2A, bands labeled with asterisks). DNA migrating in the upper part of the gel could be detected by this staining procedure (Figure 2A, lanes 8–12). The co-purification of candidate factors with the rDNA domains was investigated by western blot analyses (Figure 2B). While the LexA-CBP bait protein was present in similar amounts in all samples analyzed, the Pol I specific subunit Rpa135 was specifically detected in the 35S rDNA purification. Enrichment of the RNA polymerase I enhancer binding protein 1 (Reb1) was observed in the purification of the ARS domain containing a Pol I promoter-proximal Reb1-binding site supporting robust Reb1 binding *in vivo* (35). The TATA binding protein (Spt15/TBP) was present in all purifications containing rDNA consistent with its role in transcription initiation by all three RNA polymerases [reviewed in (43)]. TBP association with the ARS domain could correlate with a role for yeast TBP in DNA replication (44,45). With the exception of LexA-CBP, none of the above proteins was detected in the control purification. Taken together, interactions of non-histone proteins with the isolated rDNA domains are at least partially preserved during the purification procedure.

### Compositional analysis by comparative MS reveals distinct proteomes co-purifying with rDNA subdomains

**Figure 3.**
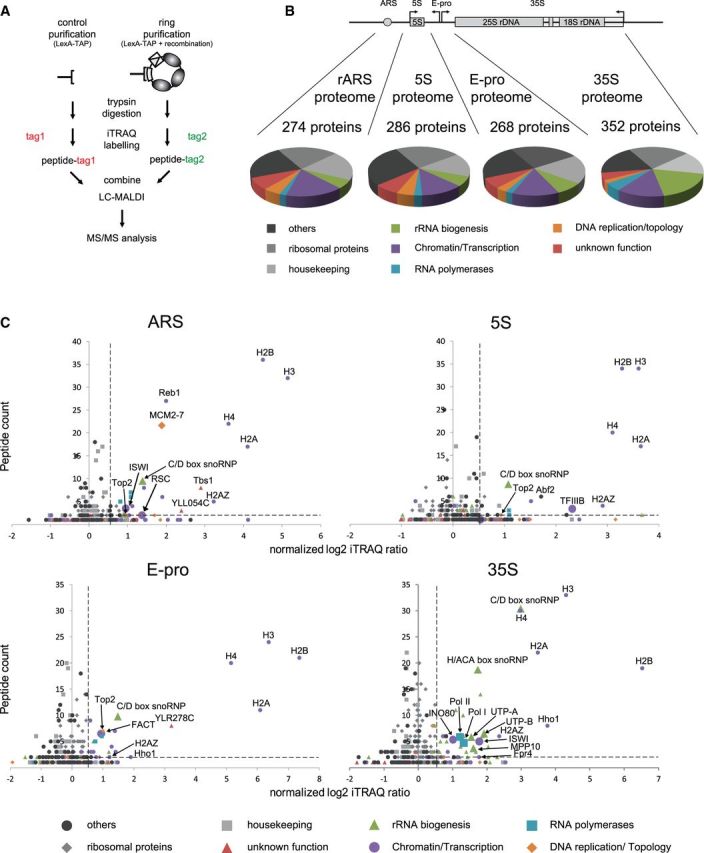
Compositional analysis by comparative MS reveals distinct proteomes co-purifying with rDNA subdomains. (**A**) Strategy of the comparative MS approach. (**B**) Classification of proteins co-purifying with rDNA chromatin domains. Proteins co-purifying with LexA-TAP from yeast strains y2383 (ARS), y2379 (5S), y2384 (E-Pro) and y2381 (35S) were subjected to iTRAQ analysis in direct comparison with control purifications from yeast strain y2378 (control). Identified proteins were categorized according to their biological function. The pie charts depict the relative fraction for each protein class in the purification. The color code for different protein classes is given on the bottom. The data represent the summary of three independent biological replicates of the purification for each individual domain. (**C**) Graphical summary of enrichment of protein factors and complexes in different rDNA domain purifications. The average iTRAQ ratio for each identified protein was calculated as described in the text and plotted against the total number of specific peptides. Dashed lines frame the area of the graph depicting enriched proteins (see text for details). For protein complexes, the average iTRAQ ratio and average number of peptides of all complex components were calculated and plotted. Note the log2 scale of the *x*-axis. Small icons are used to label single proteins, whereas large icons label protein complexes. The color and shape code for different protein classes is given on the bottom.

MS and the isobaric tag for relative and absolute quantitation (iTRAQ) technology (46) were used to analyze proteins co-purifying with rDNA domains. Tryptic peptides derived from protein samples from rDNA domain or control purifications were separately labeled using two different iTRAQ reagents (Figure 3A). The relative amounts of the differentially labeled peptides in the rDNA domain purification and in the control purification were determined by LC-MALDI-TOF/TOF MS and are depicted as iTRAQ ratio in Figure 3C; and Supplementary Data Set 1 (full MS summary). Between 250 and 350 proteins were identified in each of the purifications of the four rDNA subdomains. The proteins were classified according to their biological function (Figure 3B, pie charts).

Very similar amounts of peptides from abundant housekeeping proteins and ribosomal proteins were identified in all proteomes co-purifying with rDNA subdomains and in the respective control purification (Figure 3B) likely representing background contaminants. Therefore, the iTRAQ ratio of every protein identified in the individual analyses was divided by the average iTRAQ ratio of all housekeeping proteins and ribosomal proteins. The average iTRAQ ratio for each protein was then calculated from three biological replicate experiments and plotted against the sum of peptides of the protein identified in these replicates (Figure 3C). Owing to the background correction, housekeeping proteins and ribosomal proteins form a cluster in the center of each diagram (Figure 3C, light gray squares and gray diamonds in all graphs). Only proteins with an average iTRAQ ratio of at least 1.5, identified with at least 2 peptides were considered as enriched in individual rDNA domain purifications and are discussed later in the text (Table 2 and Figure 3C, area framed by dashed lines in the diagrams). If at least 50% of multi-protein complex components fulfilled part of these criteria, the average peptide count and iTRAQ ratio for the respective complex is depicted.

**Table 2.** Protein complexes/factors specifically enriched in three biological replicates of different rDNA chromatin preparations

Group (function)	Proteins/complexes	Chromatin domain			
		ARS	5S	E-pro	35S
Histones		H2A, H2B, H3, H4, H2AZ	H2A, H2B, H3, H4, H2AZ	H2A, H2B, H3, H4, H2AZ, Hho1	H2A, H2B, H3, H4, H2AZ, Hho1
DNA replication/topology	MCM2-7 complex	**Mcm2, Mcm3, Mcm4, Mcm5, Mcm6, Mcm7**			Mcm6^a^
	Topoisomerase	Top2	Top2	Top2	Top2^b^
	others	Smc5, Smc6			Smc5, Smc6^a^
Chromatin remodeling	RSC complex	**Htl1**^a^**, Npl6**^a^**, Rsc1**^a^**, Rsc58**^a^, **Rsc6**, **Rsc8**, **Sfh1**, **Sth1**, **Arp7**^a^, **Act1**^b^		Rsc58, Rsc6, Rsc8	Rsc8
	ISW1 complex	**Isw1, Ioc2**			**Isw1, Ioc2**^b^**, Ioc3**^a^
	INO80 complex	Rvb1, Rvb2, Arp4	Rvb2	Ies3, Rvb1, Rvb2	**Ino80, Ies1**^b^**, Ies2**^a^**, Ies3**^a^**, Rvb1, Rvb2, Arp4, Arp8**^b^
Transcription factors/complexes	FACT	Pob3		**Spt16, Pob3**	
	TFIIIB		**Bdp1, Brf1**^a^**, Spt15**		
	others	Reb1, Stb4, Spt5, Hal9			
RNA polymerases	Pol I				**Rpa190, Rpa135, Rpa49, Rpa34, Rpa12**
	Pol II	Rpo21, Rpb2	Rpo21	Rpo21	**Rpo21, Rpb2, Rpb3**^b^**, Rpb4**
	common Pol I/Pol III				Rpc19^a^, Rpc40
	common all	Rpo26	Rpo26		Rpb5, Rpb8, Rpo26, Rpb10
rRNA biogenesis	UTP-A/t-UTP				**Nan1, Utp4, Utp5, Utp8, Utp9, Utp10, Utp15**
	UTP-B/Pwp2				**Utp6, Utp13, Utp21**
	MPP10				**Mpp10, Imp3, Imp4**
	C/D box snoRNP	**Nop1, Nop56, Nop58, Snu13**	**Nop1, Nop56, Nop58, Snu13**	**Nop1, Nop56, Nop58, Snu13**	**Nop1, Nop56, Nop58, Snu13, Rrp9**
	H/ACA box snoRNP	**Cbf5, Gar1, Nhp2**		Cbf5, Gar1	**Cbf5, Gar1, Nhp2, Nop10**
	others		Dbp10		Bfr2, Emg1, Enp1, Kre33, Krr1, Prp43, Rok1, Sof1, Bud21, Dip2, Nop14, Utp22, Dbp10, Ebp2, Erb1, Has1, Nop12, Nop6, Rrp5, Rrs1, Srp40
Unknown function		Tbs1, Yll054c	Ylr241w	Ylr278c	
Others		Rpl40a, Yku80	Dnl4, Lat1, Abf2, Vps1, Crn1	Rpp1a, Pab1, Hsl1, Sum1	Fpr3, Tif2, Fpr4, Pab1, Yra1, Sum1, Yhb1, Mss116

Only proteins with an average iTRAQ ratio of at least 1.5, identified with at least 2 peptides are depicted. If 50% of multi-protein complex components were identified, identified subunits are highlighted in bold and additional proteins are depicted.

^a^Identified with an average iTRAQ ratio of at least 1.5 and 1 peptide.

^b^Identified with an average iTRAQ ratio greater than one and at least two peptides.

Distinct proteomes co-purified with the individual rDNA domains. Histones, including Htz1, the yeast homolog of H2A.Z, were equally enriched in all preparations correlating with the SDS–PAGE analysis (Figure 2A). The yeast homolog of the linker histone H1, Hho1, instead was preferentially found in E-pro and 35S rDNA domain preparations. This result agrees with a previously reported role for Hho1 in 35S rDNA chromatin structure (47,48). The MCM2-7 complex, the predominant replicative helicase [reviewed in (49)], was selectively identified in the ARS domain preparation, as was Reb1 in good agreement with the western blot analysis (Figure 2B). The Spt15/TBP-containing Pol III initiation complex TFIIIB co-purified with the 5S rDNA domain in good agreement with the results in Figure 2B (panel Spt15/TBP). In the 35S rDNA chromatin preparation, 11 of 14 subunits of Pol I were identified. Unexpectedly, 8 of 12 subunits of Pol II were also detected in this sample. Pol II has been shown to transcribe rDNA in some conditions (50–54) but might artificially associate with this domain during purification (see Discussion). In the 35S rDNA preparation, a specific group of ribosome biogenesis factors including constituents of the H/ACA box and C/D box snoRNPs were identified. These proteins are thought to bind co-transcriptionally to nascent rRNAs extending from elongating Pol I (55–58). Although C/D box snoRNPs were strongly enriched in the 35S rDNA preparation, they were also detected in all other rDNA purifications, which could point to a more general function of these complexes or be explained by the presence of higher-order recombination products including 35S rDNA (see Figure 1D and Supplementary Figure S1) in these preparations. Different chromatin remodeling complexes co-purified with individual rDNA domains. Whereas 10 of 17 subunits of the RSC complex were detected in the ARS domain purification, many subunits of the INO80 chromatin remodeling complex co-purified with the 35S rDNA. ISW1 complex components were enriched in ARS and 35S rDNA preparations, corroborating the reported interaction of this complex with the rDNA locus *in vivo* (59,60). The main components of a Pol II elongation factor, the FACT complex, were present in the E-pro domain purification. In summary, for many of the factors identified by MS *in vivo* interaction with the respective domain have been reported, validating our approach. Finally, many proteins with unknown function in rDNA biology were also identified in these purifications (Table 2).

### Two uncharacterized proteins, Tbs1 and Ylr278c, and the INO80 complex associate with rDNA chromatin *in vivo*

**Figure 4.**
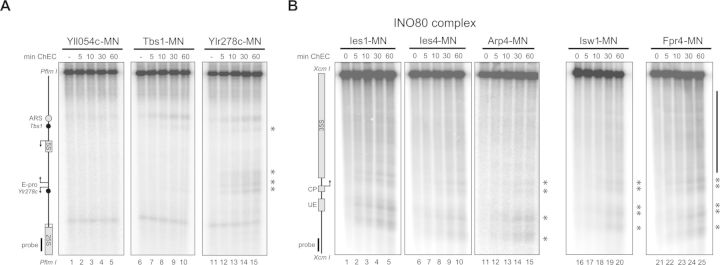
Two uncharacterized proteins, Tbs1 and Ylr278c and the INO80 complex associate with rDNA chromatin *in vivo*. (**A** and **B**) ChEC analysis with yeast strains y2707 (Yll054c-MN), y2633 (Tbs1-MN), y2634 (Ylr278c-MN), y2259 (Isw1-MN), y2157 (Ies1-MN), y2158 (Ies4-MN), y2159 (Arp4-MN) and y2258 (Fpr4-MN) expressing the indicated MNase fusion proteins from the endogenous location. Yeast strains were grown at 30°C in yeast peptone dextrose to exponential phase and treated with formaldehyde. Nuclei were prepared and incubated in the absence (−) or presence of calcium for the times indicated on top of each panel. DNA was isolated, digested with the restriction enzyme endonucleases PflmI (A), or XcmI (B) and subjected to indirect endlabeling Southern blot analysis with radioactively labeled probes rDNA_IGS (A), or rDNp (B). A cartoon of the genomic region analyzed is depicted on the left. The position of consensus sequences for binding of Tbs1 and Ylr278c within the IGS are marked as black dots. Asterisks on the right label specific MN-fusion protein-mediated cleavage events.

We arbitrarily chose three proteins co-purifying with the ARS or E-Pro domains, Yll054c, Tbs1 or Ylr278c, for which interaction with rDNA had not been previously reported. To verify their interaction with rDNA *in vivo* we used ChEC analysis (61), which has been used in the past to identify many protein–rDNA interactions (26,35). Yeast cells expressing the endogenous factors fused to micrococcal nuclease (MNase) were crosslinked with formaldehyde. Nuclei were prepared, and calcium was added to induce cleavage by the MNase fusion proteins, which can be mapped to the DNA sequence by indirect end-labeling Southern blot analysis with a probe detecting a fragment containing the IGS of the rDNA locus. While Yll054c-MNase did not cleave within this rDNA region (Figure 4, lanes 1–5), weak Tbs1-MNase dependent cleavage within the ARS region could be detected (Figure 4C, lanes 6–10, marked by asterisks). ChEC analysis with Ylr278c-MNase expressing strains also showed cleavage within the ARS region and a relatively strong cleavage within the E-pro region (Figure 4A, lanes 11–15). Tbs1 and Ylr278c belong to a class of yeast transcription factors bearing a Zn(II)2Cys6 binuclear cluster DNA binding motif (62). A consensus sequence for Ylr278c (63) and a recognition sequence for Tbs1 (63,64) containing one mismatch are located within the E-Pro and ARS regions, respectively. ChEC was also performed with yeast cells expressing MNase fusion proteins of the Ies1, Ies4 and Arp4 subunits of the INO80 complex, the Isw1 protein and the Fpr4 protein (Figure 4B). Isw1 and Fpr4, for which rDNA interaction had been reported (59,60,65), were were found to be enriched in 35S rDNA chromatin preparations and included as controls in these analyses. Southern blot analysis was performed with a probe detecting a fragment containing a large part of the 35S rDNA including the Pol I promoter region (Figure 4B). For all MNase fusion proteins, we observed cleavage within this sequence. Some cleavage mediated by the MNase fusion proteins was also observed outside of the 35S rDNA (Supplementary Figure S3). We note that MNase fusion protein mediated cleavage was weak but specific in all cases, probably indicating that these factors associate with a subpopulation of rDNA repeats. These results support the established rDNA interactions of the Isw1 and Fpr4 proteins and suggest that the INO80 complex and two proteins with yet unknown function interact with rDNA chromatin *in vivo*, indicating that the native purification approach is suitable to detect novel chromatin components.

### Major structural features of 5S rRNA gene chromatin are preserved upon isolation

**Figure 5.**
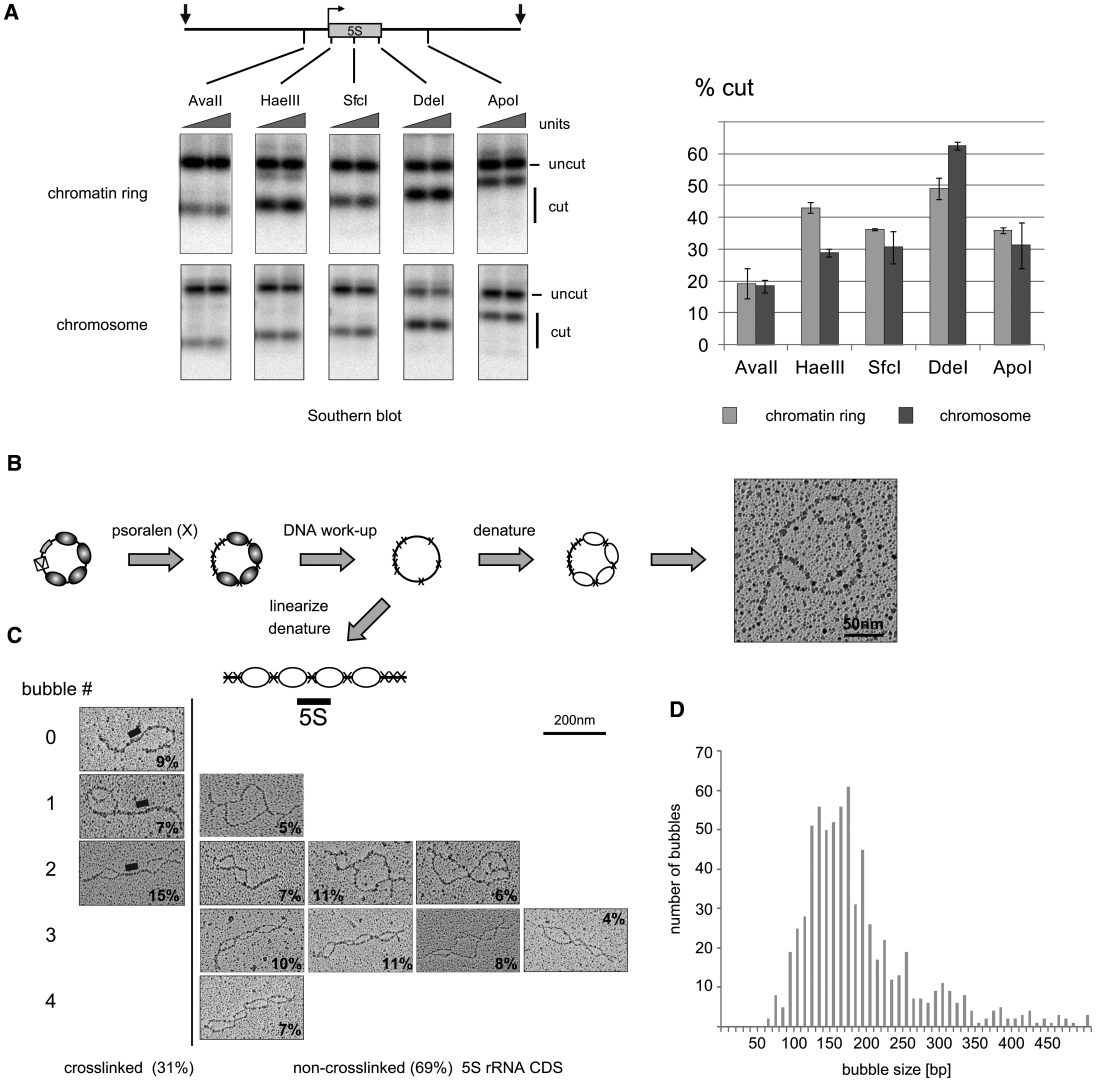
Nucleosomal protection and nucleosome positions at 5S rDNA. (**A**) Restriction endonuclease accessibilities in purified and chromosomal 5S rDNA chromatin. Purified 5S rDNA chromatin and isolated nuclei from yeast strains y1997[K2049], and y2124 or y1599, respectively, were digested with increasing amounts of the indicated restriction enzymes (triangle on top of each pair of panels). DNA was isolated, digested with NcoI (chromatin ring) or PvuI/SphI (chromosome) and subjected to indirect endlabeling Southern blot analysis with the radioactively labeled probe 5S_2. Top: schematic representation of the 5S rRNA gene locus with restriction sites used to probe chromatin structure (black lines) and restriction sites of the secondary digestion (black arrows). The positions of uncut and cut fragments are shown on the right. The histogram shows the results of Southern blot quantification as a percentage of DNA cut at the highest restriction enzyme concentration. Average and standard deviations are from two independent biological replicates. (**B–D**) Determination of nucleosome positions at the 5S rRNA gene by single molecule EM analysis. (B) Isolated chromatin rings were subjected to psoralen crosslinking (black crosses). After DNA isolation, crosslinked molecules were relaxed with the nicking endonuclease Nt.AlwI, denatured and analyzed by EM under denaturing conditions. The panel on the right shows a representative electron micrograph. (C) After linearization with the restriction endonuclease NcoI before denaturation and EM analysis, 334 molecules were analyzed and categorized according to number (given on the left), size and position of the observed single-stranded bubbles. Representative electron micrographs for molecules of each class are shown. The percentage of each class in the total population of molecules is depicted in the lower right corner of the micrographs. The left-most electron micrographs show three classes containing a crosslinked 5S rRNA coding sequence (position indicated by a black bar in each electron micrograph). (D) The bar graph depicts bubble size distribution (in bp) of 701 single-stranded DNA bubbles measured in the total population of 334 molecules.

Structural integrity of the 5S rDNA domain was verified by treating purified chromatin or the chromosomal 5S rRNA gene in yeast nuclei with restriction enzymes. Samples were treated with five different enzymes with recognition sites within the 5S rRNA gene sequence and in the flanking regions, respectively (Figure 5A). DNA was isolated and analyzed by Southern blot. Overall restriction enzyme DNA accessibilities were similar in the isolated chromatin domain and on the chromosome in isolated yeast nuclei (Figure 5A, compare upper and lower panel on the left, see graph on the right for quantitation). We conclude that major structural properties of 5S rDNA chromatin are preserved upon purification.

### EM analysis determines nucleosome positions at 5S rDNA

Restriction enzyme accessibility allows evaluating the average nucleosomal protection against nuclease attack in a population of chromatin rings. Additionally, nucleosome occupancies and positions on single chromatin molecules could be visualized by EM (66,67). Isolated 5S rDNA rings were crosslinked with psoralen. Psoralen intercalates into double-stranded nucleic acids and establishes covalent crosslinks between DNA strands upon irradiation with long wave ultraviolet light. DNA assembled into a nucleosome is protected from psoralen incorporation leaving a footprint of the nucleosome of ∼150 bp of uncrosslinked DNA (Figure 5B). DNA isolated from psoralen treated purified 5S rDNA was analyzed by EM under denaturing conditions. In these analyses, 5S rDNA molecules appeared as a circle with defined crosslinked double-stranded regions and single-stranded DNA bubbles (see Figure 5B electron micrograph on the right; 150 bp scale to ∼50 nm).

DNA was linearized such that the 5S rRNA gene was in the center of the molecule to map the positions of the observed single-stranded DNA bubbles (Figure 5C). A total of 334 linear DNA molecules were evaluated and classified into 12 different groups according to number, position and size of single-stranded DNA bubbles. None of these classes was strongly overrepresented indicating marked heterogeneity in chromatin structure within the region flanking and encompassing the 5S rRNA gene. Analysis of bubble size showed that 57% of the bubbles had either a size expected for the protection by one nucleosome (130–180 bp) or two nucleosomes (280–360 bp) (Figure 5D). Around 26% of the DNA bubbles had an intermediate size between 180 and 260 bp, which might be the consequence of incomplete crosslinking of naked DNA due to sequence preferences for psoralen-crosslinking, or protection by chromatin components other than nucleosomes. The 5S rRNA coding sequence was psoralen crosslinked in three different molecule classes representing 31% of the analyzed molecules (Figure 5C, left panels, position of the 5S rRNA gene marked by a black bar), which likely represented the nucleosome-free transcriptional active state of the gene (68). These results are in good agreement with the restriction enzyme accessibility in the purified chromatin domain, where 33–36% of the molecules are cut by SfcI in the center of the 5S rRNA coding sequence (Figure 5A) and with the observation that up to one-third of the 5S rRNA genes on the chromosome are actively transcribed (69).

### MS identifies proteins co-purifying with the single copy *PHO5* gene

**Figure 6.**
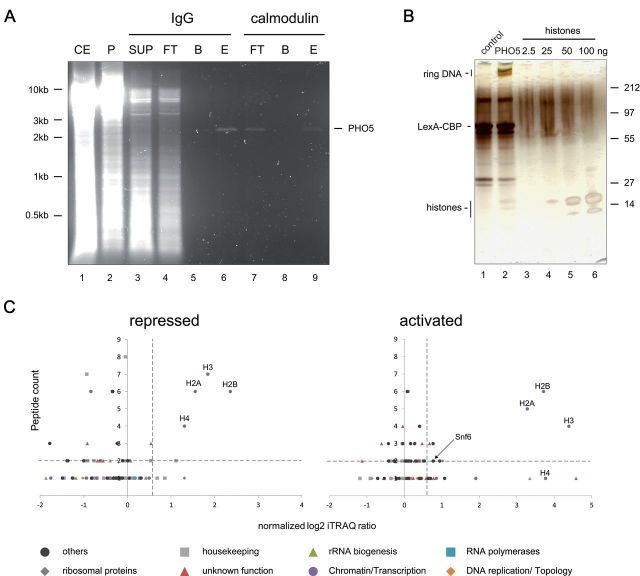
MS identifies proteins co-purifying with the single copy *PHO5* gene. (**A**) DNA analysis of samples of a TAP of *PHO5* gene rings. Yeast strain y2629 carrying a *PHO5* locus flanked by RS elements was subjected to TAP. DNA was isolated from samples CE, SUP, P and FT, E, and B from IgG and calmodulin affinity purifications, digested with NcoI and analyzed as described in the legend to Figure 1C. Chromatin was purified from 1.5 × 10^12^ cells and 0.01% (CE), 0.02% (SUP, P, FT), 2.5% (B, E; IgG, FT; calmodulin) and 3.3% (B, E, lanes 8–9; calmodulin) from the respective sample were analyzed. Positions of DNA size markers and of the NcoI fragment of the *PHO5* gene ring are indicated. (**B**) Analysis of proteins co-purifying with *PHO5* chromatin domains. Proteins co-purifying from calmodulin sepharose with LexA-CBP from yeast strains y2629 (*PHO5*, see above) and y2628 (control) carrying an unmodified *PHO5* locus were separated in an SDS–PAGE gradient gel (4–12%) stained with silver. The LexA bait protein was purified from ∼1.5 × 10^12^ cells, and 95% of the respective samples were used for analysis. For relative quantification, defined amounts of recombinant human histone octamers were analyzed in the same gel. Positions of protein size markers, and LexA-CBP, the core histones, as well as the ring DNA (visualized by silver stain) are indicated. (**C**) Graphical summary of enrichment of proteins in *PHO5* gene ring purifications. Proteins co-purifying with *PHO5* gene rings isolated from cells in which *PHO5* transcription was repressed (y464[K2048]), or constitutively activated (y465[K2048]) were subjected to iTRAQ analysis in direct comparison with purifications of the corresponding control strains (y454[K2048], or y455[K2048], respectively). Data of two independent replicates were evaluated as described in the legend to Figure 3.

To test whether the approach is suitable to purify and analyze chromatin from a single copy locus, *PHO5* gene chromatin was isolated from a strain in which the gene was tagged with a cluster of LexA-binding sites and flanked by RS elements. To limit background contaminations, a second affinity purification step using calmodulin sepharose was introduced. DNA analysis of different samples of the TAP revealed a single DNA band at the expected size for the linearized *PHO5* gene ring in the final elution (Figure 6A, lane 9). Around 50 fmoles of purified ring DNA could be obtained from ten liters of exponentially growing yeast culture (∼10^12^ cells) corresponding to a recovery of ∼3% of total *PHO5* loci (Table 1). In the final elution fraction of the TAP, the specific chromosomal domain was >100 000-fold enriched over another single copy locus (Table 1).

Equal enrichment of the LexA-CBP bait proteins in the final elution of the control and *PHO5* chromatin preparation was verified by SDS–PAGE and silver staining (Figure 6B). Proteins migrating with the mobility of histone proteins were detected in the sample containing the purified *PHO5* domain but not in the control purification (Figure 6B, lanes 1 and 2). The amount of histone proteins co-purifying with the *PHO5* ring correlated well with the expectation from the amount of ring DNA analyzed in the gel. The protein composition of purified *PHO5* gene chromatin was further analyzed by semiquantitative MS comparing ring purifications with control purifications. Chromatin rings were isolated from two different strains in which transcription of the *PHO5* gene was either repressed or constitutively activated by the deletion of the *PHO80* gene (70,71). Transcriptional activation leads to drastic structural changes in *PHO5* promoter chromatin (72,73). In good accordance with the results of the SDS–PAGE analysis, canonical histone proteins were specifically enriched in all *PHO5* chromatin purifications in comparison with purifications from the respective control strains lacking RS elements and LexA-binding sites (Figure 6C). Probably owing to the more stringent purification conditions, only a few proteins besides the canonical histones met our criteria for enrichment in these purifications. One of these proteins Snf6, a subunit of the SWI/SNF chromatin remodeling complex, co-purified specifically with the activated *PHO5* chromatin (Figure 6C and Supplementary Data Set 2 for full MS summary).

### Snf6 associates with the activated *PHO5* promoter region *in vivo*

**Figure 7.**
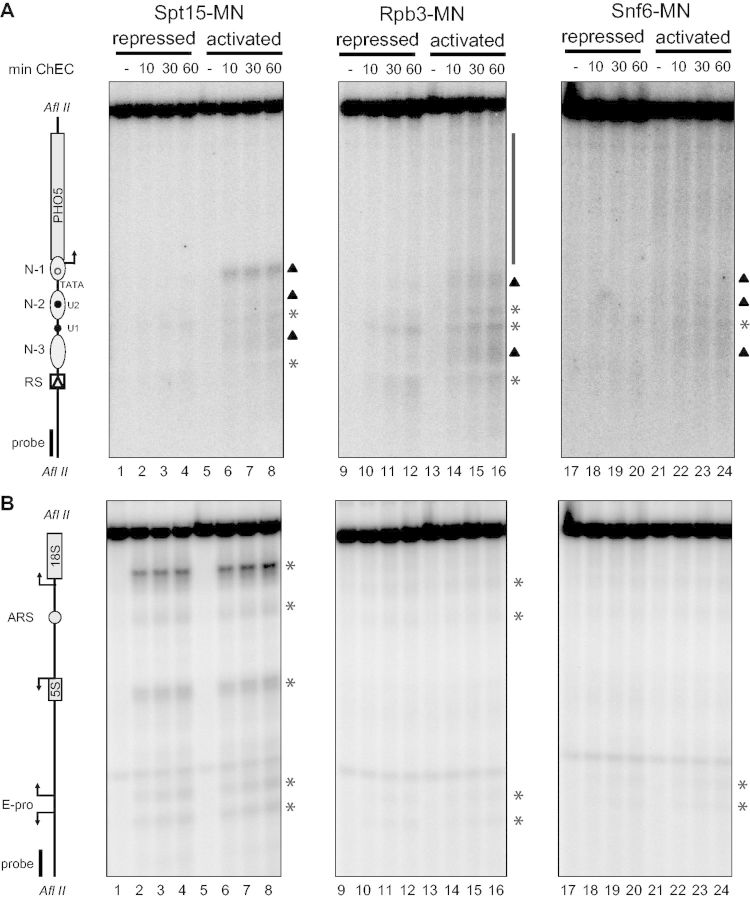
Snf6 associates with the activated *PHO5* promoter region *in vivo*. (**A** and **B**) ChEC analysis was performed as described in the legend to Figure 4 with yeast strains y2970 (Spt15-MN, repressed), y2971 (Spt15-MN, activated), y2972 (Rpb3-MN, repressed), y2973 (Rpb3-MN, activated), y2978 (Snf6-MN, repressed) or y2979 (Snf6-MN, activated) and subjected to indirect endlabeling Southern blot analysis with radioactively labeled probes PHO5 (A) or ENH_RFB (B). A cartoon of the genomic regions analyzed is depicted on the left. Asterisks on the right mark specific MN-fusion protein mediated cleavage events in transcriptionally repressed and active *PHO5* gene chromatin, as well as in the IGS region of the rDNA, whereas triangles mark cleavage events specifically occurring in transcriptionally active *PHO5* gene chromatin.

SWI/SNF has been reported to play a role in the activation of *PHO5* expression on phosphate starvation (74–76), as well as mitotic induction of the gene (77). ChEC was used to investigate Snf6-MNase-mediated cleavage in the parental yeast strains used for the purification of the *PHO5* chromatin domains being repressed or constitutively activated in transcription. We also created strains expressing TBP/Spt15 and a subunit of RNA Pol II, Rpb3, as MNase fusion proteins. After ChEC, isolated DNA was analyzed by Southern blot analysis using a probe detecting a fragment containing the *PHO5* locus (Figure 7A). Significant TBP/Spt15-MNase-mediated cleavage events at the *PHO5* promoter were observed in the strain carrying the constitutive active gene, whereas only little cleavage was observed in the strain in which *PHO5* expression was repressed (Figure 7A, compare lanes 1–4 with 5–8, asterisks mark cleavage events at the transcriptionally repressed and active *PHO5* locus, whereas triangles label cleavage events specifically detected at the transcriptionally active *PHO5* locus). This was in good agreement with ChEC results in TBP/Spt15-MNase-expressing strains when *PHO5* gene expression was induced upon phosphate starvation (78). A very similar picture was obtained for Rpb3-MNase with cleavage events extending into the coding sequence (CDS) of the constitutively active *PHO5* gene (Figure 7A, compare lanes 9–12 with 13–16, a bar marks the cleavage events within the CDS). Enhanced interaction of TBP/Spt15 and Rbp3 with the *PHO5* locus correlates well with its activation in the *PHO80* deletion strain. Finally, weak cleavage events mediated by Snf6-MN were detected mainly within the *PHO5* promoter region in conditions when the gene was actively transcribed, whereas very limited cleavage occurred when *PHO5* transcription was repressed (Figure 7A, compare lanes 17–20 with lanes 21–24). As a control, the same membrane was subsequently hybridized with a probe detecting a fragment containing the IGS of the rDNA (Figure 7B). Cleavage events were observed for all of the MNase fusion proteins within this region. In contrast to the results obtained at the *PHO5* locus, the MNase fusion proteins cleaved to a similar extent within the IGS region, regardless if *PHO5* expression had been activated or repressed (Figure 7B compare lanes 1–4, 9–12, and 17–20 with lanes 5–8, 13–16, and 21–24, respectively). As observed earlier (35), TBP/Spt15-MNase dependent cleavage events could be observed at the Pol I promoter, the Pol III promoter, E-Pro and the ARS region (Figure 7B, lanes 1–8), consistent with the results of the western blot analysis (Figure 2B). Rpb3-MNase and Snf6-MNase-mediated cleavage was mainly restricted to the E-pro region, with additional weak cleavage events of Rbp3-MN at the ARS and at a site directly upstream of the Pol I promoter (Figure 7B, lanes 9–16 and 17–24).

Taken together, we conclude that the analysis of histone molecules and the identification of chromatin components at many single copy genomic loci in yeast might be feasible building on the herein described purification protocol.

## DISCUSSION

The possibility to purify distinct chromosomal domains under native conditions from yeast provides a solid basis to investigate chromatin biology. Similar approaches reported so far are restricted to chromatin on plasmids (15,16), which might not fully reflect the situation on a chromosome. Other attempts to derive information about chromatin composition at defined genomic locations made use of chemical crosslinking (12–14), which prevents subsequent structural or functional analyses of the isolated chromatin. Thus, the work presented here represents an important step towards the detailed characterization of native chromatin on a molecular level.

As described in the introduction, the current technique is partly based on previous work (7). Although the earlier study provided evidence that major features of chromatin isolated from a single copy locus were preserved on purification, the purified material did not allow for the determination of the protein composition because of high background contaminations (unpublished results). Furthermore, the identification of histone modifications and structural analysis of the purified material remained unachieved. Eventually, these analyses were only possible by rigorous modification of the earlier protocol, which involved creation of a large set of yeast strains each carrying genomic modifications allowing site-specific recombination at a distinct target locus. In addition, various yeast integration vectors were constructed to optimize endogenous expression levels of the recombinant LexA-TAP protein. This led to a substantial reduction of chromatin fragments, that were non-specifically enriched in the presence of excess LexA-TAP [data not shown and (16)]. Other important improvements were to partially adapt a protocol, which has been used for the isolation of ribonucleoprotein complexes in the past, and to turn to comparative MS to define specifically co-purifying proteomes. Finally, the successful purification of chromatin domains from the multi-copy rDNA locus helped defining the amounts of material needed for the isolation of chromatin from a single-copy gene locus that would be amenable to MS and EM analyses.

Chemical crosslinking has been used to stabilize chromatin upon purification (12–14). In a previous study, Pol I or Pol II were isolated from cells after crosslinking the proteins to their chromatin template, and co-purifying proteomes were compared by semiquantitative MS (58). As the only essential function of yeast Pol I is the transcription of the 35S rRNA gene (79), the Pol I proteome likely represents actively transcribed 35S rRNA gene chromatin, which is largely depleted of nucleosomes (26). Strikingly, there was a strong overlap between the Pol I enriched proteome under crosslinking conditions and the proteome co-purifying with the native 35S rDNA domain [compare Table 2 with Figure 6B and C in (58)]. Amongst others, a large set of early acting ribosome biogenesis factors was identified in both studies, indicating that even certain co-transcriptional assembling ribonucleoprotein complexes (55–58) partly persist throughout the native purification procedure. Some of these proteins had been previously identified as part of a nucleolar complex co-purifying with Pol I (80). In summary, only a few proteins enriched in the purification of chromatin fragments crosslinked to Pol I were not present in native 35S rDNA domain preparations (compare Table 2 with Figure 6B and C in (58)). In contrast, purified 35S rDNA chromatin additionally contained non-transcribed nucleosomal, rRNA gene chromatin evident from enrichment of histone proteins and chromatin remodeling complexes, which were either depleted or not present in the chromatin preparation crosslinking to Pol I (58). Thus, chemical crosslink might not be a necessity to preserve many of the protein DNA interactions in chromatin.

One of the few unexpected findings was that a significant subset of specific Pol II subunits co-purified with the 35S rDNA domain. It has been previously reported that the formation of extrachromosomal ribosomal circles in respiratory deficient yeast strains leads to synthesis of rRNA by Pol II instead of Pol I (50). The formation of extrachromosomal ribosomal circle resembles the release of rRNA genes by site-specific recombination used for isolation of the chromatin domains in this study. Thus, it is possible that rDNA recombination events are linked to Pol II recruitment to this locus. However, ChIP experiments did not support a direct correlation between rDNA circle generation by R recombinase and association of Pol II (Supplementary Figure S3). Another explanation for the aforementioned observation could be that Pol II can access the rDNA locus on the chromosome to some extent in normal conditions. Pol II transcription was suggested to occur at different locations of rDNA, originating from the E-Pro region (81), a cryptic promoter within IGS2 (39), or a Pol II dependent gene located antisense within the 25S rDNA region [reviewed in (82)]. Additionally, there is evidence that Pol II can transcribe nucleosomal rRNA genes, which are not transcribed by Pol I (83). Thus, mass-spectrometric analysis might be sensitive enough to detect even a limited number of Pol II molecules associated with rDNA, and it cannot be excluded that the purification protocol partly selects for the subpopulation of genes transcribed by Pol II. Alternatively, Pol II association with the 35S rDNA domain may occur as an artifact of the purification at a step downstream of the recombination event.

EM analysis of DNA derived from psoralen treated rDNA chromatin purified from exponentially growing cells provided detailed information about nucleosome occupancy and positioning on the 5S rRNA gene sequence. Future research will aim to correlate different nucleosomal configurations with the transcriptional state of this locus. Comparative EM analyses of the single copy *PHO5* gene in a transcriptionally activated and repressed state have thus provided further clues to elucidate the mechanism of transcriptional activation (see “note added in proof” for C.B. and H.B., accepted manuscript). Furthermore, structural analysis of purified chromatin under native conditions is likely to enhance our understanding of the structure-function relationship of genes in defined functional states.

Besides the benefit to obtain compositional and structural information, the purified native chromatin provides a highly defined template for *in vitro* experiments likely reflecting the *in vivo* situation with regard to nucleosome positioning and histone modification. This can be an advantage over the use of artificial, *in vitro* reconstituted nucleosomal arrays. Along these lines, purified chromatin of transcriptionally activated and repressed *PHO5* genes has already been subjected to *in vitro* manipulation (7,84,85).

## NOTE ADDED IN PROOF

The accepted manuscript by C.B. and H.B. mentioned in the Discussion section can be found under: Brown,C.R., Mao,C., Falkovskaia,E., Jurica,M.S. and Boeger,H. (2013) Linking stochastic fluctuations in chromatin structure and gene expression. PLoS Biol., 11, e1001621. While this manuscript was under revision other manuscripts reporting MS analyses of purified chromatin domains have been published which might be interesting for scientists in the field: Neumüller,R.A., Gross,T., Samsonova,A.A., Vinayagam,A., Buckner,M., Founk,K., Hu,Y., Sharifpoor,S., Rosebrock,A.P., Andrews,B., et al. (2013) Conserved regulators of nucleolar size revealed by global phenotypic analyses. Sci. Signal., 6, ra70; Fujita,T. and Fujii,H. (2013) Efficient isolation of specific genomic regions and identification of associated proteins by engineered DNA-binding molecule-mediated chromatin immunoprecipitation (enChIP) using CRISPR. Biochem. Biophys. Res. Commun., 439, 132–136.; Byrum,S.D., Taverna,S.D. and Tackett,A.J. (2013) Purification of a specific native genomic locus for proteomic analysis. Nucleic Acids Res., 10.1093/nar/gkt822; Pourfarzad,F., Aghajanirefah,A., de Boer,E., Ten Have,S., Bryn van Dijk,T., Kheradmandkia,S., Stadhouders,R., Thongjuea,S., Soler,E., Gillemans,N., et al. (2013) Locus-Specific Proteomics by TChP: Targeted Chromatin Purification. Cell Reports, 4, 589–600.

## SUPPLEMENTARY DATA

Supplementary Data are available at NAR Online, including [86–90].

## FUNDING

German Research Foundation (DFG) in the context of the SFB960 (to H.T., P.M., and J.G.). Funding for open access charge: DFG programme “Open Access Publishing” [INST 89/318-3].

*Conflict of interest statement*. None declared.

## Supplementary Material

Supplementary Data
