# Supplementary material for: Compositional and structural analysis of selected chromosomal domains from Saccharomyces cerevisiae
Source: Nucleic Acids Res. 2013 Oct 6;42(1):e2. doi: 10.1093/nar/gkt891 (PMC3874202; doi:10.1093/nar/gkt891)
Supplement: Supplementary Data [file supp_42_1_e2__index.html]

Compositional and structural analysis of selected chromosomal domains from Saccharomyces cerevisiae — Compositional and structural analysis of selected chromosomal domains from Saccharomyces cerevisiae — Supplementary Data 

# Compositional and structural analysis of selected chromosomal domains from *Saccharomyces cerevisiae*

## Supplementary Data

files

**Files in this Data Supplement:**

- Supplementary Data - pdf file
- Supplementary Data - xlsx file
- Supplementary Data - xlsx file
