# Supplementary material for: Compositional and structural analysis of selected chromosomal domains from Saccharomyces cerevisiae
Source: Nucleic Acids Res. 2013 Oct 6;42(1):e2. doi: 10.1093/nar/gkt891 (PMC3874202; doi:10.1093/nar/gkt891)
Supplement: Supplementary Data [file supp_gkt891_nar-01140-met-k-2013-File009.pdf]

Compositional and structural analysis of selected chromosomal domains from *Saccharomyces cerevisiae*

Stephan Hamperl[1,$], Christopher Brown[3], Ana Villar Garea[1], Jorge Perez-Fernandez[1], Astrid Bruckmann[1], Katharina Huber[1], Manuel Wittner[1,$$], Virginia Babl[1], Ulrike Stoeckl[1], Rainer Deutzmann[1], Hinrich Boeger[3], Herbert Tschochner[1,#], Philipp Milkereit[1,#], and Joachim Griesenbeck[1,#]

[1] Universität Regensburg, Biochemie-Zentrum Regensburg (BZR), 93053 Regensburg, Germany

[2] Department of Molecular, Cell, and Developmental Biology, University of California Santa Cruz, Santa Cruz, CA 95064, USA

[$] present address: Chemical and Systems Biology, Stanford University, CA 94305-5441, USA

[$$] present address: Boehringer Ingelheim Pharma GmbH, 88397 Biberach/ Riß, Germany

Supplementary Data

Supplementary Datasets 2

Supplementary Dataset 1: 2

Supplementary Dataset 2: 2

Supplementary Figures and Tables 4

Supplementary Figure 1 (related to Figure 1): 4

Supplementary Figure 2 (related to Figure 4) 6

Supplementary Figure 3 8

Supplementary Table 1: Oligonucleotides used in this study 10

Supplementary Table 2: Plasmids used in this study 13

Supplementary Table 3: Yeast strains used in this study 14

Supplementary Table 4: Antibodies used in this study 15

Supplementary Table 5: Southern probes used in this study 16

Supplementary References 17

Supplementary Datasets

Supplementary Dataset 1:

Detailed results of mass spectrometric analyses of three independent purifications of of the ARS, 5S, E-Pro and 35S rDNA chromatin domains.

LC-Maldi TOF/TOF iTRAQ analyses of purified samples of the respective rDNA domain were carried out in direct comparison with a purified sample of a control strain using the Applied Biosystems 4800 Proteomics Analyzer MALDI TOF/TOF mass spectrometer. The Microsoft Excel file includes the original MS_MS_summary datasets generated by searching the NCBInr protein sequence database with the Mascot search engine (Matrix Science) implemented in the GPS Explorer software (Applied Biosystems) for each the 3 individual purifications of the four individual domains (35S_#1-3_MSMS_summary, ARS_#1-3_MSMS_summary, 5S_#1-3_MSMS_summary and E-pro_#1-3_MSMS_summary). It further contains three spreadsheets for each of the 3 individual purifications of the four individual domains summarizing the MS-MS Data (35S_#1-3, ARS_#1-3, 5_#1-3S and E-pro_#1-3). Only peptides with an ion score confidence interval (c.i.) of more than 95% were included in these spreadsheets, and the following information is provided: Common gene name; Systematic gene name; Accession number; Protein classification as detailed in the Excel worksheet “class_definition”; Peptide count, Average iTRAQ ratio, and iTRAQ Standard Deviation extracted from the original MS_MS_summary sheets; Protein localization, Protein copy number and Protein description (86, 87); http://www.yeastgenome.org). For each individual domain the datasets of the three independent purifications was combined in one spreadsheet (Summary_35S, Summary_ARS, Summary_5S, Summary_E-Pro) providing the following information: Main class/common gene name; normalized average iTRAQ ratio; Sum of Peptide Count. The normalized average iTRAQ ratio was calculated from the quotient of the iTRAQ ratio of each protein divided by the average iTRAQ ratio of the groups of ribosomal proteins and housekeeping proteins (main class A1, A2, G) in the independent purifications. The spreadsheet was used to generate the scatter plots and builds the basis for Table 2.

Supplementary Dataset 2:

Detailed results of LC-Maldi TOF/TOF iTRAQ analyses of two independent purifications of PHO5 gene rings from cells in which PHO5 transcription is either repressed (PHO5(r)) or constitutively activated (PHO5(a)).

LC-Maldi TOF/TOF iTRAQ analyses of purified samples of the respective *PHO5* domain were carried out in direct comparison with a purified sample of a control strain using the Applied Biosystems 4800 Proteomics Analyzer MALDI TOF/TOF mass spectrometer. Data

from two independent purifications is presented as detailed in the legend to Supplementary Dataset 1.

Supplementary Figures and Tables

Supplementary Figure 1 (related to Figure 1):

DNA analysis of samples of the purification of rDNA subdomains

A-D) Yeast strains y2267 (ARS) (A), y2379 (5S) (B), y2268 (E-pro) (C) and y2381 (35S) (D), carrying the indicated rDNA chromatin domain flanked by RS sites, were subjected to the purification procedure. DNA was isolated from samples (CE, SUP, P, FT, and E, see Figure 1A) and separated in a 1% agarose gel either after digestion with NcoI (A-C) or with SacII (D) (lanes 1-12) or without digestion (lanes 13-18). DNA was visualized by SYBR® Safe staining (lanes 1-6) or in a Southern blot analysis (lanes 7-18) with the radioactively labeled probes ARS (A), 5S_1 (B), E-pro (C) or 18S (D) (see Supplementary Table 8). Positions of DNA size markers and of the linearized (lanes 1-12) or nicked and supercoiled (lanes 13-18) ring DNA are given on the left and on the right, respectively. Asterisks mark fragments obtained from higher-order recombination products containing the respective rDNA segment. Purifications were performed from 10^{11} cells and 0.2% (CE), 0.3% (SUP, P, FT) and 2% (E, B) (lanes 1-12) or 0.1% (CE), 0.15% (SUP, P, FT) and 1% (E, B) (lanes 13-18) of the respective samples were analyzed.

The amounts of the specific rDNA subdomains were quantified using a titration series of plasmid K365 (21), linearized with NcoI, separated in the same agarose gel as the analytical samples and analyzed in the Southern blot (not shown).

Supplementary Figure 1

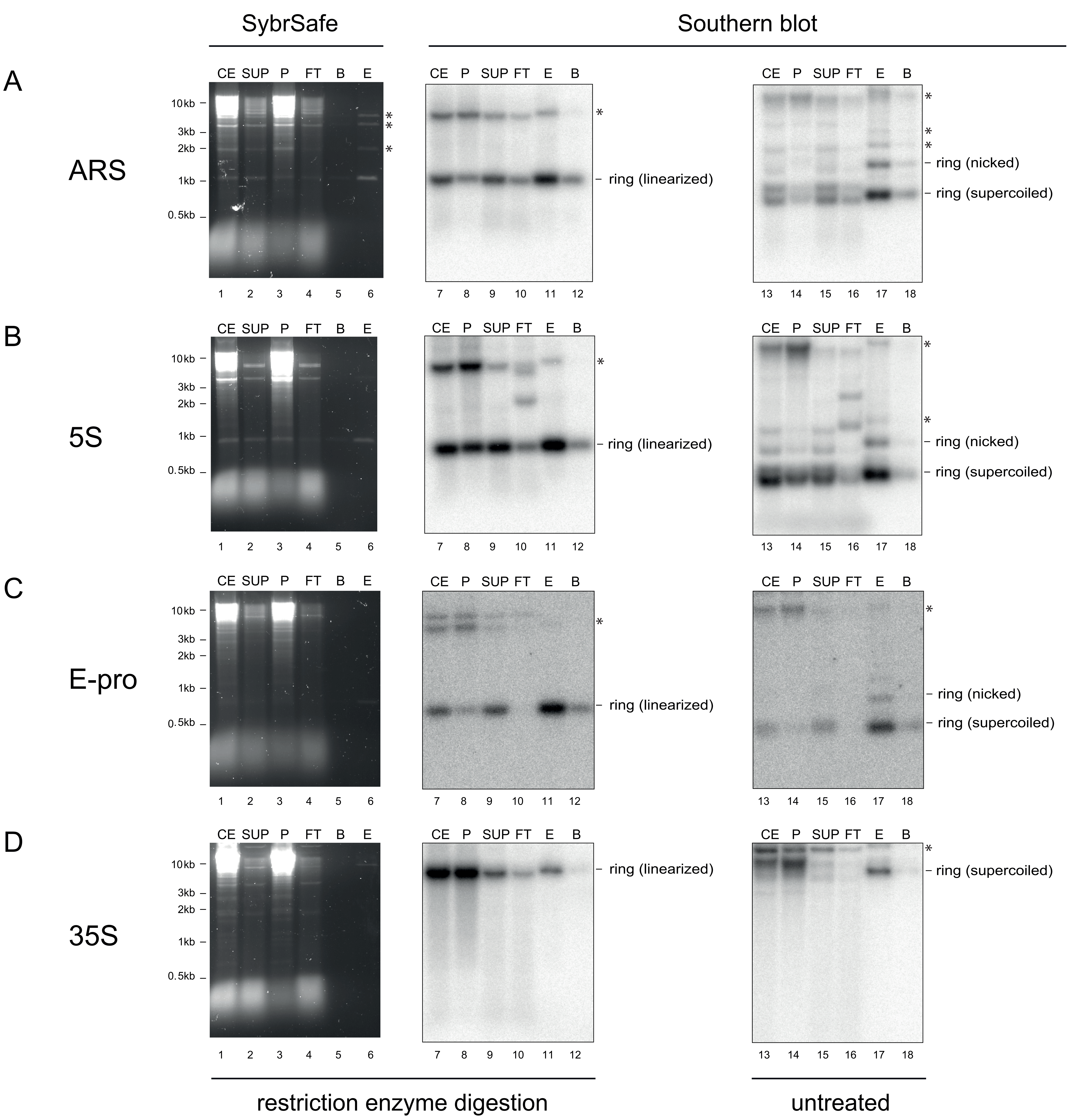

Supplementary Figure 2 (related to Figure 4)

The INO80 complex associates with rDNA chromatin in vivo
ChEC analysis with yeast strains y2259 (Isw1-MN), y2157 (Ies1-MN), y2158 (Ies4-MN), y2159 (Arp4-MN), and y2258 (Fpr4-MN) expressing MNase fusion proteins indicated on top of each panel. ChEC experiments were performed as described in the legend to Figure 4. DNA was isolated, digested with the restriction enzyme endonucleases XcmI subjected to indirect end-labeling Southern blot analysis with radioactively labeled probes IGS2. Cartoons of the genomic regions analyzed are depicted on the left. Asterisks and black lines on the right label specific MN-fusion protein mediated cleavage events as detailed in the text.

Supplementary Figure 2

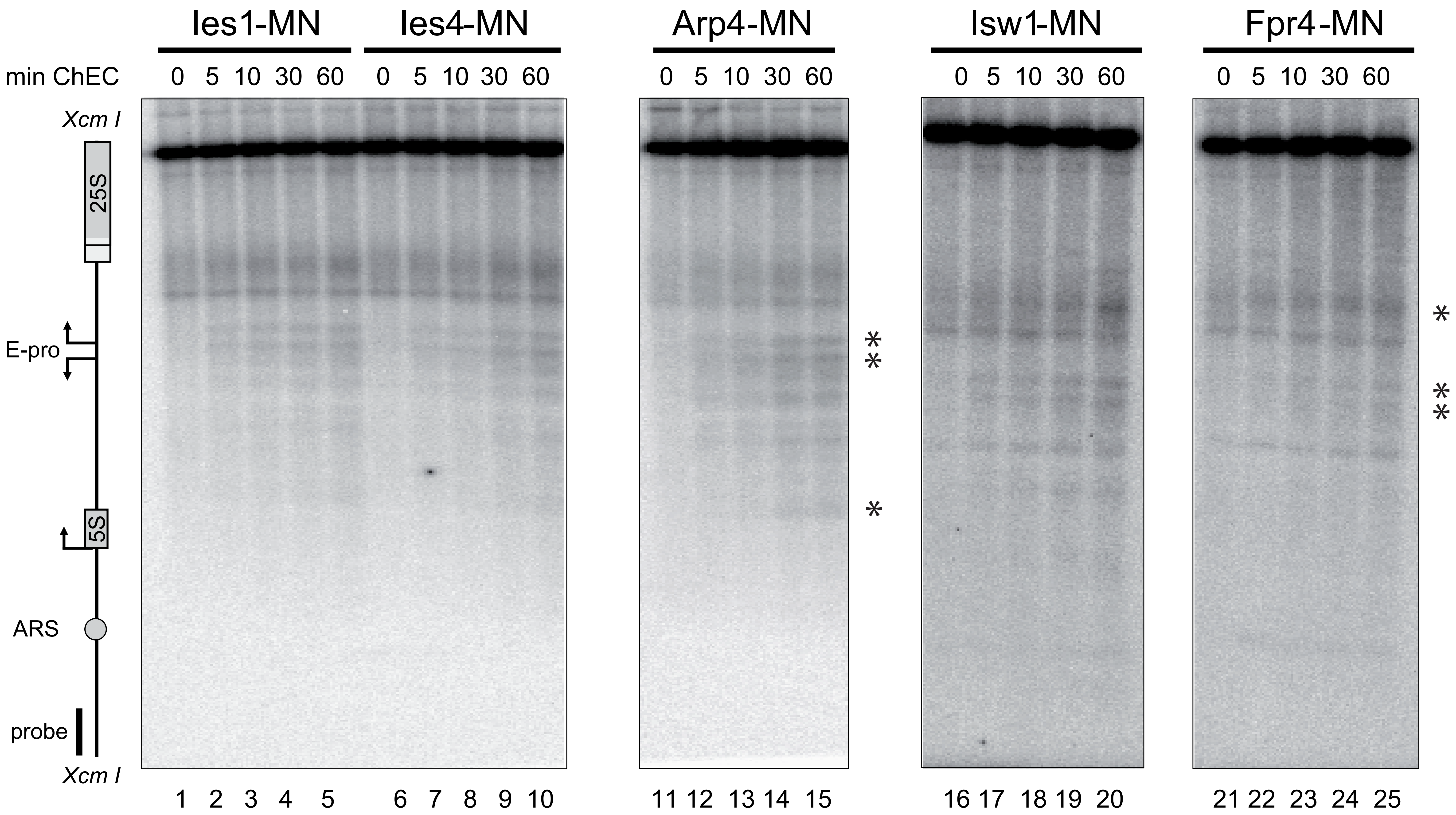

Supplementary Figure 3

Recombination of the 35S rDNA domain from chromosome XII has no significant impact on the association of Pol I and Pol II at the rDNA locus in vivo

Yeast strains y2842 (35S circle) and y2843 (control), expressing Rpa135 (Pol I) as fusion protein with a triple HA-tag or y2844 (35S circle) and y2845 (control) expressing Rpb3 (Pol II) as fusion protein with a triple HA-tag, were cultured in raffinose containing medium and exponentially growing cells were split in two equal cultures. Recombination was induced by addition of galactose to a final concentration of 2% in one of the cultures and incubation of both cultures was continued for further 1.5h before treatment with formaldehyde. ChIP experiments were performed as described (35). A) Cartoon of the yeast rDNA locus on chromosome XII shows the position of DNA regions analyzed by quantitative PCR (amplicons 1-10). In addition, DNA regions transcribed by Pol II including the coding sequence (CDS) of *PDC1*, *RPS23A*, or the promoter regions of *PHO5* and *GAL1/GAL10* were included in the analysis. B) The graphs depict the percentage of the input of the respective DNA fragment co-precipitating with the HA-tagged fusion protein (Rpa135-HA or Rpb3-HA) with and without induction of recombination in the 35S rDNA circle strain or control strain as indicated. Average and standard deviation errors are derived from three independent ChIP experiments, each analyzed in triplicate qPCRs.

Co-precipitation of rDNA fragments with Rpb3-HA was low in all conditions, whereas efficient co-precipitation was observed for fragments of known Pol II transcribed loci elsewhere in the genome. In contrast Rpa135-HA co-purified substantial amounts of 35S rDNA fragments, but – as expected - only background levels of the intergenic spacer regions or Pol II transcribed loci. Therefore, increased Pol II association with rDNA as a consequence of recombination at this locus cannot explain the significant enrichment of peptides of Pol II subunits in 35S rDNA chromatin preparations in the MS-analysis (Fig. 3C, Supplementary Dataset 1).

Supplementary Figure 3

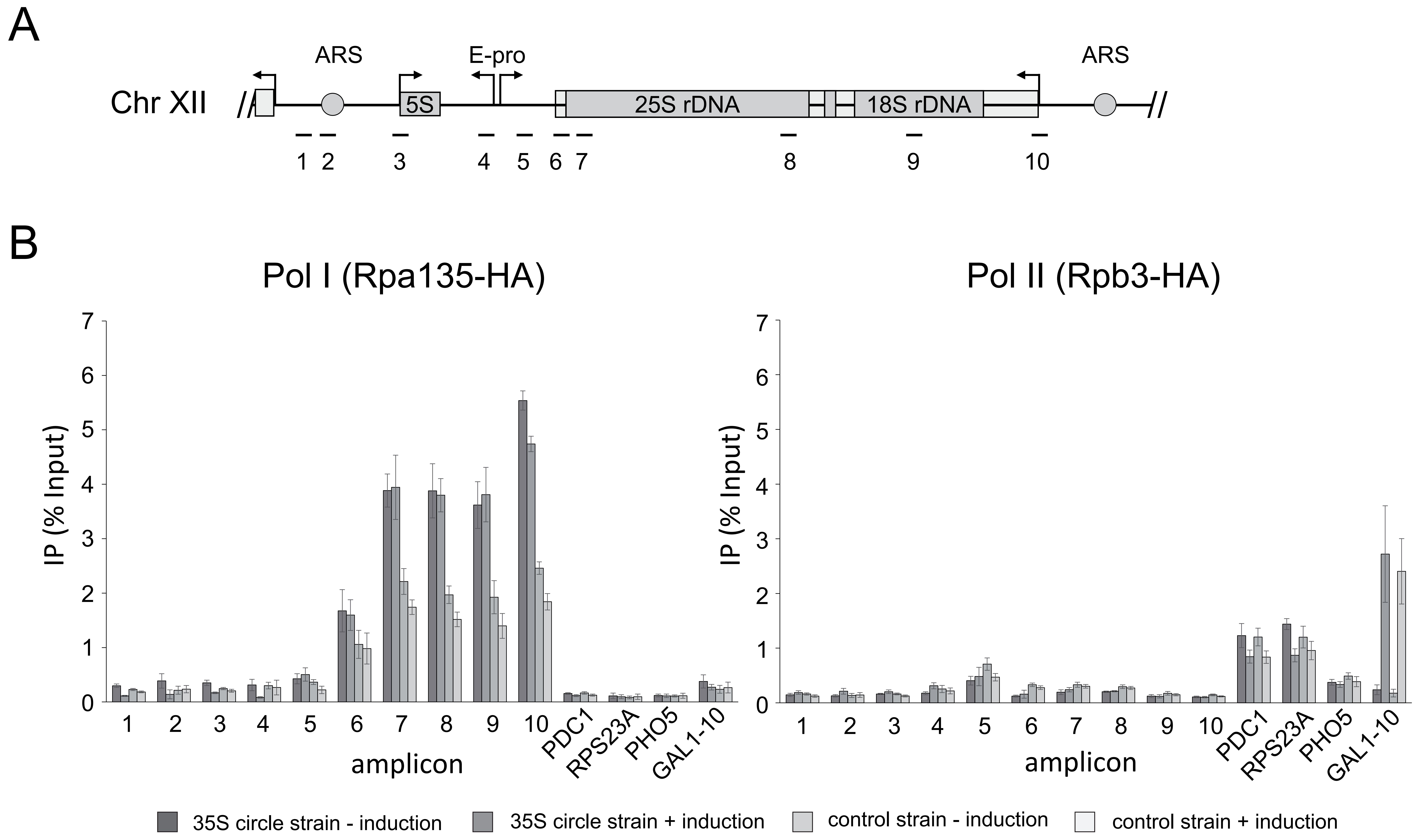

Supplementary Table 1: Oligonucleotides used in this study

#	Sequence	Purpose
613	CATGATCAGATGGGGCTTGA	Primer used for qPCR amplifying a region of the PDC1 CDS together with 614 (amplicon PDC1)
614	ACCGGTGGTAGCGACTCTGT	Primer used for qPCR amplifying a region of the PDC1 CDS together with 613 (amplicon PDC1)
688	TCATCTTATGTGCGCTGCT	Primer used for qPCR amplifying a region of the PHO5 promoter together with 689 (amplicon PHO5)
689	CAGTTGCGTTCTATGCGAAA	Primer used for qPCR amplifying a region of the PHO5 promoter together with 688 (amplicon PHO5)
710	TGGAGCAAAGAAATCACCGC	Primer used for qPCR amplifying a region in 25S rDNA together with 711 (apmlicon 7)
711	CCGCTGGATTATGGCTGAAC	Primer used to obtain template for Southern probe preparation from yeast genomic DNA together with 1558 and used for qPCR amplifying a region in 25S rDNA together with 710 (amplicon 7)
712	GAGTCCTTGTGGCTCTTGGC	Primer used for qPCR amplifying a region in 18S rDNA together with 713 (amplicon 9)
713	GAGTCCTTGTGGCTCTTGGC	Primer used for qPCR amplifying a region in 18S rDNA together with 712 (amplicon 9)
817	GAGGGACGGTTGAAAGTG	Primer used to obtain template for Southern probe preparation from yeast genomic DNA together with 818
818	ATACGCTTCAGAGACCCTAA	Primer used to obtain template for Southern probe preparation from yeast genomic DNA together with 817
920	GCCATATCTACCAGAAAGCACC	Primer used for qPCR amplifying a region in 5S rDNA together with 921 (amplicon 3)
921	GATTGCAGCACCTGAGTTTCG	Primer used for qPCR amplifying a region in 5S rDNA together with 920 (amplicon 3)
969	TCATGGAGTACAAGTGTGAGGA	Primer used for qPCR amplifying a region in 35S rDNA promoter together with 970 (amplicon 10)
970	TAACGAACGACAAGCCTACTC	Primer used for qPCR amplifying a region in 35S rDNA promoter together with 969 (amplicon 10)
1045	TTAAATTGATGAAAGAATACGTTATTCTTTCATCAAGTTAACGTGAAGGTGTCA TTCGCAG	Primer used for oligo annealing together with 1046 to insert a HpaII-RS site in pT28 AflII site
1046	TTAAATTGATGAAAGAATACGTTATTCTTTCATCAAGTTAACGTGAAGGTGTCA TTCGCAG	Primer used for oligo annealing together with 1045 to insert a HpaII-RS site in pT28 AflII site
1161	CAGGTTATGAAGATATGGTGCAA	Primer used to obtain template for Southern probe preparation from yeast genomic DNA together with 1162 and used for qPCR amplifying a region in rARS region together with 1162 (amplicon 2)
1162	AAAATGGCCTATCGGAATACA	Primer used to obtain template for Southern probe preparation from yeast genomic DNA together with 1161 and used for qPCR amplifying a region in rARS region together with 1161 (amplicon 2)
1167	TGGATCTAATTTACAGCAGCA	Primer used to obtain template for Southern probe preparation from yeast genomic DNA together with 1168
1168	CCTGATCCCACTCTTCTTGA	Primer used to obtain template for Southern probe preparation from yeast genomic DNA together with 1167
1342	GATGGTACCCCAAGCTTTTGAAGCTATATACCCTGTGCTAAGTGAATTTAGAA AAATGTCGTACGCTGCAGGTCGAC	Primer used to obtain amplicon from K643 for genomic integration of MNase-3xHA::KanMX6 of SPT15 together with 1343
1343	CTACCGCGGAAATGGAACAAATAGAAAACCTTTTTTTCTTTTCGTCTACTCCTT CCCCAATCGATGAATTCGAGCTCG	Primer used to obtain amplicon from K643 for genomic integration of MNase-3xHA::KanMX6 of SPT15 together with 1342
1351	TTTACTTCGCCAACCATTCC	Primer used to obtain template for Southern probe preparation from yeast genomic DNA together with 2590
1510	TCTCCATTCGGTGGTTCTTC	Primer used for qPCR amplifying a region of RPS23A CDS together with 1511 (amplicon RPS23A)
1511	ATCGTTTGGAACGAAAGCAG	Primer used for qPCR amplifying a region of RPS23A CDS together with 1510 (amplicon

		RPS23A)
1558	ATGATTTATCCCCACGCAAA	Primer used to obtain template for Southern probe preparation from yeast genomic DNA together with 711
1859	CGATGGAAGTTTGAGGCAAT	Primer used to obtain template for Southern probe preparation from yeast genomic DNA together with 1860
1860	GTACAAAGGGCAGGGACGTA	Primer used to obtain template for Southern probe preparation from yeast genomic DNA together with 1859
2099	TGTCCTCCACCCATAACACC	Primer used to obtain template for Southern probe preparation from yeast genomic DNA together with 2100
2100	ATTTAGCATAGGAAGCCAAG	Primer used to obtain template for Southern probe preparation from yeast genomic DNA together with 2099
2293	GTTCCGCGGTGCATCTCAAATGGGTAATACTGGATCAGGAGGGTATGATAAT GCTTGGTCGTACGCTGCAGGTCGAC	Primer used to obtain amplicon from K643 for genomic integration of MNase-3xHA::KanMX6 of RPB3 together with 2294
2294	GTTGGTACCTTTTCGGTTCGTTCACTTGTTTTTTTTCTTCTATTACGCCCACTT GAGAAATCGATGAATTCGAGCTCG	Primer used to obtain amplicon from K643 for genomic integration of MNase-3xHA::KanMX6 of RPB3 together with 2293
2419	GGAATGGTTGGCGAAGTAAA	Primer used for qPCR amplifying a region of E-pro together with 2420 (amplicon 4)
2420	TCCCTCCATTTCCCTCTCTT	Primer used for qPCR amplifying a region of E-pro together with 2419 (amplicon 4)
2494	GATGGTACCACGCGCGGAAATTGATGACGAAGAAGCAACCGCCATGTTTAAG CTGGAGTCGTACGCTGCAGGTCGAC	Primer used to obtain amplicon from K643 for genomic integration of MNase-3xHA::KanMX6 of IES1 together with 2495
2495	CTACCGCGGTTCTTAAATGTATGTATGTGTGTGTGTGTGTGTGCGTATTGT TCTATTATCGATGAATTCGAGCTCG	Primer used to obtain amplicon from K643 for genomic integration of Mnase-3xHA::KanMX6 of IES1 together with 2494
2496	GATGGTACCTCCTGAAGTAAAACAACTTGAAAAAGAAGGAGAGGATGGACTG GACTCATCGTACGCTGCAGGTCGAC	Primer used to obtain amplicon from K643 for genomic integration of Mnase-3xHA::KanMX6 of IES4 together with 2497
2497	CTACCGCGGAACTAGAAAGTGTGGGGCCCTGAGAACAACTTTAAGCTGTTGA CATTACCATCGATGAATTCGAGCTCG	Primer used to obtain amplicon from K643 for genomic integration of Mnase-3xHA::KanMX6 of IES4 together with 2496
2498	GATGGTACCGGAATACGAAGAGGTGGGCGTCGAAAGATTGCTTAACGATAGG TTTAGATCGTACGCTGCAGGTCGAC	Primer used to obtain amplicon from K643 for genomic integration of Mnase-3xHA::KanMX6 of ARP4 together with 2499
2499	CTACCGCGGCAAACTGCTAAACTGAAAGGCGACTTGTCATTCAACAACGTTTT CTATTCATCGATGAATTCGAGCTCG	Primer used to obtain amplicon from K643 for genomic integration of Mnase-3xHA::KanMX6 of ARP4 together with 2498
2507	CTGGAGCTCGGATCCCAAAGCGCCAGTTCATTTGGCG	Primer used to obtain amplicon of CYC1 promoter fragment from yeast genomic DNA together with 2511
2508	CTGGAGCTCGGATCCCACCCAGACCGCGACAAATTA	Primer used to obtain amplicon of TEF2 promoter fragment from yeast genomic DNA together with 2512
2511	AGTTCTAGAGTGTTTGTGTGTCTATAGAAG	Primer used to obtain amplicon of CYC1 promoter fragment from yeast genomic DNA together with 2507
2512	AGTTCTAGACGTTGACCGTATATTCTAAAA	Primer used to obtain amplicon of TEF2 promoter fragment from yeast genomic DNA together with 2508
2590	AGCGTCAAAGGATGAGGCTA	Primer used to obtain template for Southern probe preparation from yeast genomic DNA together with 1351
2629	TGGCGGCCGCCAGC	5' phosphorylated self-complementary oligonucleotide with central NotI cleavage site and SacII compatible 3' overhang
2663	GATGGTACCTCCTGCTAACTCTGAATTGACATTTGATGTTAAATTGGTCTCCAT GAAATCGTACGCTGCAGGTCGAC	Primer used to obtain amplicon from K643 for genomic integration of MNase-3xHA::KanMX6 of FPR4 together with 2672
2665	GATGGTACCAACTGAACAGTTGGTAGCAGAGAAAATTCCGGAAAACGAAACC ACTCATTCGTACGCTGCAGGTCGAC	Primer used to obtain amplicon from K643 for genomic integration of MNase-3xHA::KanMX6 of ISW1 together with 2674
2672	CTACCGCGGTTGTATATAGTATTATAGATACATATATCAATACGTATGCATTAA GGACCATCGATGAATTCGAGCTCG	Primer used to obtain amplicon from K643 for genomic integration of MNase-3xHA::KanMX6 of FPR4 together with 2663
2674	CTACCGCGGAGCATGGTGTAGGATATATTAAAAAAAATCGAAATATAAAAAAA GAAGGTATCGATGAATTCGAGCTCG	Primer used to obtain amplicon from K643 for genomic integration of MNase-3xHA::KanMX6 of ISW1 together with 2665

2686	GATGGTACCCCTGCAGGATGTCGAAAGCTACATATAA	Primer used to obtain amplicon of URA3 gene from yeast genomic DNA together with 2687
2687	CTACCGCGGCCTGCAGGTTAGTTTTGCTGGCCGCATCT	Primer used to obtain amplicon of URA3 gene from yeast genomic DNA together with 2686
2692	TTGGATGGACGCAAAGAAGT	Primer used for qPCR amplifying a region of GAL1-10 promoter together with 2693 (amplicon GAL1-10)
2693	GGCGGCTTCTAATCCGTACT	Primer used for qPCR amplifying a region of GAL1-10 promoter together with 2692 (amplicon GAL1-10)
2811	GATGCACTTAAGAATTCTATGATCCGGGTAAAAACA	Primer used to obtain template for Southern probe preparation from yeast genomic DNA together with 2887
2864	GCATGCCTGTTTGAGCGTC	Primer used for qPCR amplifying a region of 25S rDNA together with 2865 (amplicon 8)
2865	CGACCGTACTTGCATTATACC	Primer used for qPCR amplifying a region of 25S rDNA together with 2864 (amplicon 8)
2884	TACGATGAGGATGATAGTGTGTAAGAGTG	Primer used for qPCR amplifying a region at the 3`end of 25S rDNA together with 2885 (amplicon 6)
2885	TCTCTTTTAACCCATCTTTGCAA	Primer used for qPCR amplifying a region downstream of 25S rDNA together with 2884 (amplicon 6)
2886	GAGAAAAGCTCATTTCCTATAGTTAACAG	Primer used for qPCR amplifying a region downstream of 25S rDNA together with 2887 (amplicon 5)
2887	TTCACTTGTCTCTTACATCTTTCTTGG	Primer used for qPCR amplifying a region downstream of 25S rDNA together with 2886 (amplicon 5) and to obtain template for Southern probe preparation from yeast genomic DNA together with 2811
3022	TCTGAAGCGTATTTCCGTCAC	Primer used for qPCR amplifying a region upstream of Pol I promoter together with 3023 (amplicon 1)
3023	CAACCGAAACCAAAACCAAC	Primer used for qPCR amplifying a region upstream of Pol I promoter together with 3022 (amplicon 1)
3034	CCACCTACCGACCAACTTTC	Primer used to obtain template for Southern probe preparation from yeast genomic DNA together with 3035
3035	GAGGTGTTATGGGTGGAGGA	Primer used to obtain template for Southern probe preparation from yeast genomic DNA together with 3034
3149	GATGGTACCTTTTTTTTACGATCGGGATTTCTTCTTCAAGAATGTGTGTGTAAA AATGTCGTACGCTGCAGGTCGAC	Primer used to obtain amplicon from K643 for genomic integration of MNase-3xHA::KanMX6 of TBS1 together with 3150
3150	CTACCGCGGCGCGTATGCATATGTATTAGTTAAATTACTCGAATGTCCTTTATA TAATAATCGATGAATTCGAGCTCG	Primer used to obtain amplicon from K643 for genomic integration of Mnase-3xHA::KanMX6 of TBS1 together with 3149
3243	GATGGTACCTCCGGTAGAAGACTTTTGGACGATTAATGATGACTACGGCTTTT TAACGTCGTACGCTGCAGGTCGAC	Primer used to obtain amplicon from K643 for genomic integration of Mnase-3xHA::KanMX6 of YLR278C together with 3244
3244	CTACCGCGGAAAAAAAAAAAAAAGGAATGAGTTTGTACATACTATTATATTAATG TAGTAATCGATGAATTCGAGCTCG	Primer used to obtain amplicon from K643 for genomic integration of Mnase-3xHA::KanMX6 of YLR278C together with 3243
3280	GATGGTACCAATTGATCTTGATAAGCTACTTGGAATTTTCCCTAACCTGAGTAA CTTTTCGTACGCTGCAGGTCGAC	Primer used to obtain amplicon from K643 for genomic integration of Mnase-3xHA::KanMX6 of YLL054C together with 3281
3281	CTACCGCGGAGCATTAGTTTACTAACTTTCTCCTCGTATCTTTCAAATTTGTAT TCCCCATCGATGAATTCGAGCTCG	Primer used to obtain amplicon from K643 for genomic integration of Mnase-3xHA::KanMX6 of YLL054C together with 3280
3756	GATGGTACCCAACACCAATTTCGACGATGAATTTGGAGATCTTGATGCTGTAT TTTTTTCGTACGCTGCAGGTCGAC	Primer used to obtain amplicon from K643 for genomic integration of Mnase-3xHA::KanMX6 of SNF6 together with 3757
3757	CTACCGCGGAATATGTAAAAGGAAAGTATATATTTCCAAGAAGTAGCCGCCCA TGGCTAATCGATGAATTCGAGCTCG	Primer used to obtain amplicon from K643 for genomic integration of Mnase-3xHA::KanMX6 of SNF6 together with 3756
3782	CGCAATTATTACTTGGATGCCC	Primer used to obtain template for Southern probe preparation from yeast genomic DNA together with 3783
3783	TAAGGGACCATCATCGTTAG	Primer used to obtain template for Southern probe preparation from yeast genomic DNA together with 3782

Supplementary Table 2: Plasmids used in this study

#	Name	Purpose	Origin
K322	YEPlac195	E. coli/yeast shuttle vector for expression of proteins with URA3 marker and 2µ origin of replication	(22)
K355	pB3	E. coli/yeast shuttle vector for RecR-expression under the control of the *GAL1-10* promoter.	(24)
K356	pJSS3	Yeast expression vector for LexA-TAP under control of a glyceraldehydes-phosphate dehydrogenase (GPD) promoter	(24)
K358	pM49.2	Vector used to frame genetic elements with RS sites and adding a cluster of LexA operator sites	(24)
K363	pR2	Vector used for constitutive expression of LexA-TAP under control of GPD promoter and inducible expression of R Recombinase under control of GAL1-10 promoter; LEU2 selection marker framed with RS sites	this study
K365	pT1	Vector with LEXA binding sites and RS sites flanking the whole rDNA repeat	(21)
K366	pT2	Vector with LEXA binding sites and RS sites flanking the 35S rRNA coding sequence	(21)
K368	pT4	Vector with 2xL and 1xR flanking regions of yeast rDNA locus used for cloning	(21)
K375	pT11	Vector used to construct strains with modified RDN1 locus with LEXA binding sites and RS sites flanking the whole rDNA repeat at the endogenous locus of NOY989	(21)
K389	pT25	Vector used to construct strains with modified RDN1 locus with LEXA binding sites and RS sites flanking the 35S CDS at the endogenous locus of NOY989	(21)
K643	pKM9	Vector used as template for PCRs to amplify MNase-3HA::KanMX6 cassette with primer pairs 3149/3150 and 3243/3244	(26)
K674	pT36	Vector used to construct strains with modified RDN1 locus with LEXA binding sites and RS sites flanking the 18S CDS at the endogenous locus of NOY989	this study
K773	pT28	Vector with 18S CDS of yeast rDNA locus used for cloning	(21)
K929	pKG7	Vector used for inducible expression of R Recombinase and LexA-TAP under control of bidirectional inducible GAL1-10 promoter; LEU2 selection marker framed with RS sites	this study
K1185	pAG43	Vector used to construct strains with modified RDN1 locus with LEXA binding sites and RS sites flanking the 5S CDS at the endogenous locus of NOY989	this study
K1560	pBlueScript BamHI NotI 5S	Vector with RDN1 IGS region used for cloning	this study
K1577	pUS3	Vector with RDN1 ARS region flanked with LEXA binding sites and RS sites used for cloning	this study
K1578	pUS6	Vector with RDN1 E-pro region flanked with LEXA binding sites and RS sites used for cloning	this study
K1785	pT11-WT	Vector used to construct strains with unmodified RDN1 locus at the endogenous locus of NOY989	(21)
K2024	pUS9b-NotI	Vector used for subcloning of an rDNA repeat fragment containing the E-pro region flanked by RS sites with LEXA binding sites	this study
K2025	pUS11b	E. coli /yeast shuttle vector with a modified rDNA repeat containing the E-pro region flanked by RS sites with LEXA binding sites.	this study
K2026	pUS7	Vector used to construct strains with modified RDN1 locus with LEXA binding sites and RS sites flanking the rARS at the endogenous locus of NOY989	this study
K2027	pUS12	Vector used to construct strains with modified RDN1 locus with LEXA binding sites and RS sites flanking the E-pro at the endogenous locus of NOY989	this study
K2048	pSH15	Vector used for constitutive expression of LexA-TAP under control of CYC1 promoter and inducible expression of R Recombinase under control of GAL1-10 promoter; LEU2 selection marker framed with RS sites	this study
K2049	pSH17	Vector used for constitutive expression of LexA-TAP under control of TEF2 promoter and inducible expression of R Recombinase under control of GAL1-10 promoter; LEU2 selection marker framed with RS sites	this study
K2051	pBlueScript_URA3	Vector used for genomic integration of expression cassettes in endogenous URA3 locus	this study
K2052	pSH21	Vector used for genomic integration of TEF2 LexATAP GAL1-10 RecR expression cassette by recombination in URA3 locus	this study
K2053	pSH22	Vector used for genomic integration of GAL1-10 RecR expression cassette by recombination in URA3 locus	this study
K2054	pSH23	Vector used for genomic integration of CYC1 LexATAP GAL1-10 RecR expression cassette by recombination in URA3 locus	this study

Supplementary Table 3: Yeast strains used in this study

Strain	Parent	Genotype	Reference or source
NOY989		mata ade2-1 ura3-1 trp1-1 leu2-3,112 his3-11 can1-100 rdnΔ::URA3/pNOY353	(23)
NOY505		mata ade2-1 ura3-1 trp1-1 leu2-3,112 his3-11 can1-100	(88)
yS18		matα his3-11 his3-15 leu2-3 leu2-112 ura3Δ	(89)
yM7.8	yS18	matα his3-11 his3-15 leu2-3 leu2-112 ura3Δ pho80::HIS3	(73)
yM2.1	yS18	matα his3-11 his3-15 leu2-3 leu2-112 ura3Δ *PHO5*:RS-3xLEXA-RS	(73)
yM8.14	yM2.1	matα his3-11 his3-15 leu2-3 leu2-112 ura3Δ *PHO5*:RS-3xLEXA-RS, pho80::HIS3	(73)
y908	NOY989	mata ade2-1 ura3-1 trp1-1 leu2-3,112 his3-11 can1-100 rdn-L-5S-RS-35S-3xLEXA-RS-L	this study
y909	NOY989	mata ade2-1 ura3-1 trp1-1 leu2-3,112 his3-11 can1-100 rdn-L-5S-25S-RS-18S-3xLEXA-RS-L	this study
y1599	NOY989	mata ade2-1 ura3-1 trp1-1 leu2-3,112 his3-11 can1-100 rdn-ITS1-WT	(21)
y1997	NOY989	mata ade2-1 ura3-1 trp1-1 leu2-3,112 his3-11 can1-100 rdn-L-RS-5S-LEXA-RS-35S-L	this study
y2124	NOY989	mata ade2-1 ura3-1 trp1-1 leu2-3,112 his3-11 can1-100 rdn-L-5S-35S-L	this study
y2157	NOY505	mata ade2-1 ura3-1 trp1-1 leu2-3,112 his3-11 can1-100 Ies1-MNase-3xHA-KanMX6	this study
y2158	NOY505	mata ade2-1 ura3-1 trp1-1 leu2-3,112 his3-11 can1-100 Ies4-MNase-3xHA-KanMX6	this study
y2159	NOY505	mata ade2-1 ura3-1 trp1-1 leu2-3,112 his3-11 can1-100 Arp4-MNase-3xHA-KanMX6	this study
y2160	NOY505	mata ade2-1 ura3-1 trp1-1 leu2-3,112 his3-11 can1-100 Taf14-MNase-3xHA-KanMX6	this study
y2258	NOY505	mata ade2-1 ura3-1 trp1-1 leu2-3,112 his3-11 can1-100 Fpr4-MNase-3xHA-KanMX6	this study
y2259	NOY505	mata ade2-1 ura3-1 trp1-1 leu2-3,112 his3-11 can1-100 Isw1-MNase-3xHA-KanMX6	this study
y2264	NOY505	mata ade2-1 ura3-1 trp1-1 leu2-3,112 his3-11 can1-100 Top2-MNase-3xHA-KanMX6	this study
y2267	NOY989	mata ade2-1 ura3-1 trp1-1 leu2-3,112 his3-11 can1-100 rdn-L-RS-LEXA-rARS-RS-5S-35S-L	this study
y2268	NOY989	mata ade2-1 ura3-1 trp1-1 leu2-3,112 his3-11 can1-100 rdn-L-5S-RS-E-pro-LEXA-RS-35S-L	this study
y2345	NOY989	mata ade2-1 ura3-1 trp1-1 leu2-3,112 his3-11 can1-100 rdn-L-RS-5S-35S-LEXA-RS-L	this study
y2378	y2124	mata ade2-1 ura3-1 trp1-1 leu2-3,112 his3-11 can1-100 rdn-L-5S-35S-L URA3::LEU2 pTEF2 LEXA-TAP pGAL RecR	this study
y2379	y1997	mata ade2-1 ura3-1 trp1-1 leu2-3,112 his3-11 can1-100 rdn-L-RS-5S-LEXA-RS-35S-L URA3::LEU2 pTEF2 LEXA-TAP pGAL RecR	this study
y2380	y909	mata ade2-1 ura3-1 trp1-1 leu2-3,112 his3-11 can1-100 rdn-L-5S-25S-RS-18S-3xLEXA-RS-L URA3::LEU2 pTEF2 LEXA-TAP pGAL RecR	this study
y2381	y908	mata ade2-1 ura3-1 trp1-1 leu2-3,112 his3-11 can1-100 rdn-L-5S-RS-35S-3xLEXA-RS-L URA3::LEU2 pTEF2 LEXA-TAP pGAL RecR	this study
y2382	y2345	mata ade2-1 ura3-1 trp1-1 leu2-3,112 his3-11 can1-100 rdn-L-RS-5S-35S-LEXA-RS-L URA3::LEU2 pTEF2 LEXA-TAP pGAL RecR	this study
y2383	y2267	mata ade2-1 ura3-1 trp1-1 leu2-3,112 his3-11 can1-100 rdn-L-RS-LEXA-rARS-RS-5S-35S-L URA3::LEU2 pTEF2 LEXA-TAP pGAL RecR	this study
y2384	y2268	mata ade2-1 ura3-1 trp1-1 leu2-3,112 his3-11 can1-100 rdn-L-5S-RS-E-pro-LEXA-RS-35S-L URA3::LEU2 pTEF2 LEXA-TAP pGAL RecR	this study
y2386	y908	mata ade2-1 ura3-1 trp1-1 leu2-3,112 his3-11 can1-100 rdn-L-5S-RS-35S-3xLEXA-RS-L URA3::LEU2 pGAL RecR	this study
y2388	y2124	mata ade2-1 ura3-1 trp1-1 leu2-3,112 his3-11 can1-100 rdn-L-5S-35S-L URA3::LEU2 pGAL RecR	this study
y2628	yS18	matα his3-11 his3-15 leu2-3 leu2-112 ura3Δ URA3::LEU2 pCYC1 LEXA-TAP pGAL RecR	this study
y2629	yM2.1	matα his3-11 his3-15 leu2-3 leu2-112 ura3Δ *PHO5*:RS-3xLEXA-RS URA3::LEU2 pCYC1 LEXA-TAP pGAL RecR	this study
y2633	y2388	mata ade2-1 ura3-1 trp1-1 leu2-3,112 his3-11 can1-100 rdn-L-5S-35S-L URA3::LEU2 pGAL RecR Tbs1-MNase-3xHA-KanMX6	this study
y2634	y2388	mata ade2-1 ura3-1 trp1-1 leu2-3,112 his3-11 can1-100 rdn-L-5S-35S-L URA3::LEU2 pGAL RecR Ylr278c-MNase-3xHA-KanMX6	this study
y2707	y2388	mata ade2-1 ura3-1 trp1-1 leu2-3,112 his3-11 can1-100 rdn-L-5S-35S-L URA3::LEU2 pGAL RecR Yll054c-MNase-3xHA-KanMX6	this study
y2842	y2386	mata ade2-1 ura3-1 trp1-1 leu2-3,112 his3-11 can1-100 rdn-L-5S-RS-35S-3xLEXA-RS-L URA3::LEU2 pGAL RecR Rpa135-MNase-3xHA-KANMX6	this study
y2843	y2388	mata ade2-1 ura3-1 trp1-1 leu2-3,112 his3-11 can1-100 rdn-L-5S-35S-L URA3::LEU2 pGAL RecR Rpa135-MNase-3xHA-KANMX6	this study
y2844	y2386	mata ade2-1 ura3-1 trp1-1 leu2-3,112 his3-11 can1-100 rdn-L-5S-RS-35S-3xLEXA-RS-L URA3::LEU2 pGAL RecR Rpb3-MNase-3xHA-KANMX6	this study
y2845	y2388	mata ade2-1 ura3-1 trp1-1 leu2-3,112 his3-11 can1-100 rdn-L-5S-35S-L URA3::LEU2 pGAL RecR Rpb3-MNase-3xHA-KANMX6	this study
y2970	yM2.1	matα his3-11 his3-15 leu2-3 leu2-112 ura3Δ *PHO5*:RS-3xLEXA-RS Spt15-MNase-3xHA-KANMX6	this study
y2971	yM8.14	matα his3-11 his3-15 leu2-3 leu2-112 ura3Δ *PHO5*:RS-3xLEXA-RS, pho80::HIS3 Spt15-MNase-3xHA-KANMX6	this study
y2972	yM2.1	matα his3-11 his3-15 leu2-3 leu2-112 ura3Δ *PHO5*:RS-3xLEXA-RS Rpb3-MNase-3xHA-KANMX6	this study
y2973	yM8.14	matα his3-11 his3-15 leu2-3 leu2-112 ura3Δ *PHO5*:RS-3xLEXA-RS, pho80::HIS3 Rpb3-MNase-3xHA-KANMX6	this study

y2978	yM2.1	matα his3-11 his3-15 leu2-3 leu2-112 ura3Δ *PHO5*:RS-3xLEXA-RS Snf6-MNase-3xHA-KANMX6	this study
y2979	yM8.14	matα his3-11 his3-15 leu2-3 leu2-112 ura3Δ *PHO5*:RS-3xLEXA-RS, pho80::HIS3 Snf6-MNase-3xHA-KANMX6	this study

Supplementary Table 4: Antibodies used in this study

antigen	antibody	supplier/reference	species	Dilution
CBP	P-18	Santa Cruz Biotechnology	goat	1:3000
H3	ab1791	Abcam	rabbit	1:1000
H3K4me3	ab8580	Abcam	rabbit	1:1000
H3K36me3	ab9050	Abcam	rabbit	1:1000
H4Ac	06-598	Millipore	rabbit	1:1000
Reb1	-	Kindly provided by Robert Reeder	rabbit	1:5000
Rpa135	-	(90)	rabbit	1:5000
TBP	-	Kindly provided by Roger Kornberg	rabbit	1:5000 – 1:10000

Supplementary Table 5: Southern probes used in this study

Probe/target	Synthesis	Locus	Restriction enzyme	Fragment size (kb)
5S_1	PCR from genomic DNA using primers 2099 and 2100	5S	NcoI	-
ARS	PCR from plasmid K2026 (pUS7) using primers 1161 and 1162	ARS	NcoI	-
E-pro	PCR from plasmid K2027 (pUS12) using primers 1351 and 2590	E-pro	NcoI	-
18S	PCR from yeast genomic DNA using primers 1859 and 1860	18S	SacII	-
rDNp	PCR from genomic DNA using primers 817 and 818	rDNA	XcmI	4.9
IGS2	PCR from genomic DNA using primers 1161 and 1162	rDNA	XcmI	4.3
rDNA_IGS	PCR from genomic DNA using primers 711 and 1558	rDNA	PflMI	9.2
5S_2	PCR from genomic DNA using primers 3034 and 3035	5S	NcoI<br>PvuI/SphI	0.77<br>0.74
PHO5	PCR from genomic DNA using primers 3782 and 3783	PHO5	AflII	2.7
ENH_RFB	PCR from genomic DNA using primers 2811 and 2887	rDNA	AflII/NsiI	3.1

Supplementary References

86. Ghaemmaghami,S., Huh,W.-K., Bower,K., Howson,R.W., Belle,A., Dephoure,N., O'Shea,E.K. and Weissman,J.S. (2003) Global analysis of protein expression in yeast. *Nature*, **425**, 737–741.

87. Huh,W.-K., Falvo,J.V., Gerke,L.C., Carroll,A.S., Howson,R.W., Weissman,J.S. and O'Shea,E.K. (2003) Global analysis of protein localization in budding yeast. *Nature*, **425**, 686–691.

88. Nogi,Y., Yano,R., Dodd,J., Carles,C. and Nomura,M. (1993) Gene RRN4 in Saccharomyces Cerevisiae Encodes the A12.2 Subunit of RNA Polymerase I and Is Essential Only at High Temperatures. *Mol. Cell. Biol.*, **13**, 114–122.

89. Sengstag,C. and Hinnen,A. (1987) The sequence of the Saccharomyces cerevisiae gene PH02 codes for a regulatory protein with unusual aminoacid composition. *Nucl. Acids Res.*, **15**, 233–246.

90. Buhler,J.M., Huet,J., Davies,K.E., Sentenac,A. and Fromageot,P. (1980) Immunological studies of yeast nuclear RNA polymerases at the subunit level. *J. Biol. Chem.*, **255**, 9949–9954.
